# Ribosomal allostery as a potential regulator of bacterial dormancy

**DOI:** 10.1038/s41467-026-74199-2

**Published:** 2026-06-12

**Authors:** Danis Yangaliev, Eun Chae Moon, Gürol M. Süel, S. Banu Ozkan

**Affiliations:** 1 Center for Biological Physics, Arizona State University, Tempe, AZ USA; 2 Department of Physics, Arizona State University, Tempe, AZ USA; 3 Department of Molecular Biology, School of Biological Sciences, University of California San Diego, La Jolla, CA USA

**Keywords:** Molecular conformation, Computational biophysics, Biophysical chemistry, Molecular modelling

## Abstract

Ribosomes are central to protein synthesis but also serve as dynamic hubs that integrate cellular stress responses. Here, we investigate how ribosomal protein L11 regulates ribosome conformational dynamics and long-distance coupling. Long-timescale molecular dynamics simulations of wild-type and L11-deleted (ΔL11) ribosomes reveal that L11 functions as a global allosteric regulator coordinating communication between the ribosomal stalk and the peptidyl transferase center. The absence of L11 disrupts long-distance couplings involving RelA and Obg and rigidifies the hibernation-promoting factor site, suggesting altered hibernation dynamics that could affect ribosome persistence under stress. To examine the physiological implications of these computational predictions, we construct a ΔL11 *Bacillus subtilis* strain and quantify its sporulation behavior. The ΔL11 variant exhibits delayed entry into and exit from dormancy, consistent with a breakdown in stress-adaptive ribosomal regulation. Overall, these results highlight the role of L11 in ribosomal allostery, suggesting how local perturbations propagate through the ribosome to influence global physiological outcomes and bacterial survival under environmental stress.

## Abstract (web summary)

Ribosomes are crucial for protein synthesis and managing cellular stress. Here, authors show that ribosomal protein L11 acts as a global regulator, coordinating complex internal signaling to ensure proper bacterial survival during environmental stress.

## Introduction

Ribosome is the most fundamental machinery of the cell, universally responsible for catalyzing peptide bond formation and sustaining life through protein synthesis. Decades of structural, biochemical, and theoretical work have revealed that the ribosome is far more than a passive peptide-synthesizing machine. Recent computational and experimental studies have demonstrated that the ribosome is an intrinsically allosteric network, in which motions at one functional center are transmitted across tens of nanometers to regulate the activity of distant sites^1–6^. Such long-range communication enables coordination between the peptidyl transferase center, decoding site, mRNA–tRNA binding sites, factor-binding regions, and ribosomal stalks, ensuring the speed and fidelity of translation^7–11^. Building on this foundation, recent studies have emphasized that ribosomes also serve as crucial regulators of cellular physiology^12,13^: they act as sensors of nutrient stress through RelA-mediated activation of the stringent response^14–17^, as guardians of viability during stationary phase through ribosome hibernation factors such as HPF and RMF^18–22^, and even as participants in developmental decision-making, as in the relocalization of ribosomes to the forespore during *Bacillus subtilis* sporulation^23–25^. These discoveries underscore that the ribosome is not merely the machinery of protein synthesis, but a central hub that integrates environmental cues, coordinates cellular state, and regulates survival strategies well beyond translation itself.

Despite extensive advances in understanding ribosomal structure and function, the role of ribosomal allostery in governing spore entry and revival has remained unexpectedly elusive. Here, we pursue this question to determine whether the intrinsic allosteric network of the ribosome impacts this critical survival strategy in *B. subtilis* spores. To this end, we investigate how the dynamics and allosteric networks of wild-type ribosomes differ from those of a naturally occurring ΔL11 variant^26^. Ribosomal protein L11 (or uL11 in universal nomenclature) occupies a particularly strategic position in this context: located at the base of the L7/L12 stalk, L11 is a key communication node that couples factor binding, GTPase activation, and structural transitions across the large subunit^27–30^. Classical biochemical studies have long implicated L11 in recruiting translation factors and in regulating stress responses^16,27^, but its contribution to ribosome-scale allosteric communication and developmental physiology has not been addressed. Furthermore, GTPase Obg, a master regulator of sporulation in *B. subtilis*, binds near the base of the L7/L12 stalk in close proximity to ribosomal protein L11^31–33^. Intriguingly, nature itself provides an excellent model system—certain bacterial lineages, including *B. subtilis*, harbor a viable ΔL11 ribosome variant, in which the entire L11 protein is absent. Early genetic and biochemical analyses demonstrated that these ΔL11 strains remain viable but display pleiotropic defects in growth^30^, stress adaptation, and translation factor interaction^27^. This natural ΔL11 variant offers an opportunity to explore how the absence of a single ribosomal hub rewires allosteric communication and, in turn, could impact global physiological processes such as sporulation and outgrowth. By leveraging this natural ΔL11 system, we rigorously probed the function of L11 as a linchpin of ribosomal allostery and assessed its broader role in regulating a critical bacterial survival strategy.

To study the allosteric impact of ΔL11, we employ a newly developed coarse-grained RNA force field^34^ compatible with Martini 3^35,36^, enabling extensive molecular dynamics (MD) simulations that yield a total 240 μs of MD data, capturing ribosome-scale motions inaccessible to atomistic approaches. Using this framework, we systematically map the dynamics and allosteric communication pathways of both wild-type and ΔL11 ribosomes. These simulations reveal that loss of L11 profoundly remodels the ribosome’s allosteric network, disrupting correlated motions between key functional centers and weakening long-range coupling required for regulatory control. Notably, ΔL11 ribosomes display altered 100S dimer dynamics: the hibernation-promoting factor (HPF) binding site becomes rigidified and decoupled from the rest of the particle, consistent with a breakdown in ribosome dimerization and persistence. To assess the predicted impact of ΔL11 ribosomes on bacterial dormancy, we study entry and exit from spore state in a ΔL11 strain. We find that both sporulation and outgrowth are impaired, and together, these results suggest that dismantling a single ribosomal hub not only collapses ribosome-scale allostery but may also compromise survival strategies that span cellular scales, pointing to a potential role for ribosomal regulation in sporulation.

## Results

### Computational part

**Fig. 1:**
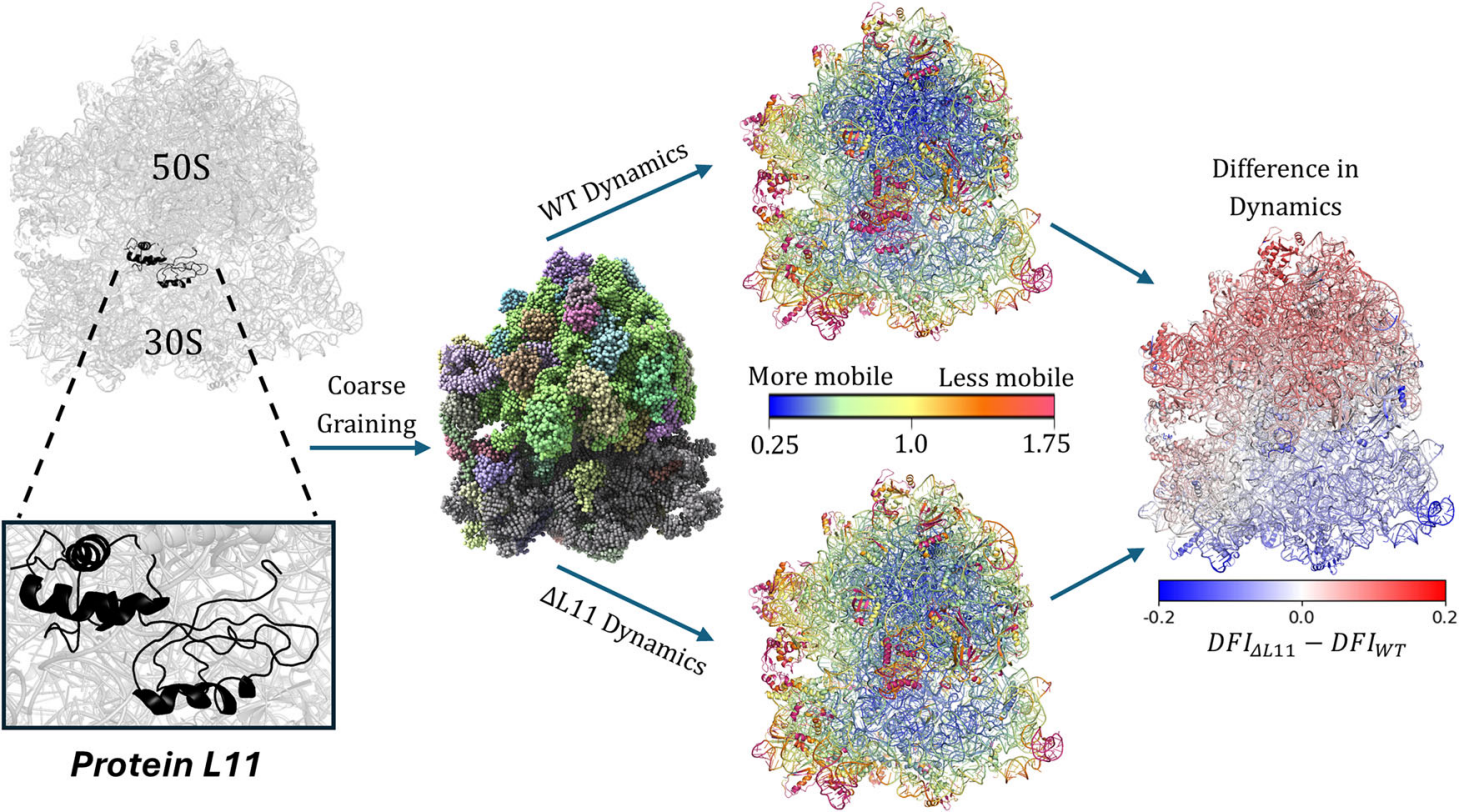
Workflow for analyzing large-scale ribosome dynamics using coarse-grained molecular dynamics simulations*.* The atomic structure of the 70S ribosome (left) is coarse-grained using the Martini 3 force field to generate CG models of the wild-type (WT) ribosome and a mutant lacking the L11 protein (ΔL11). CGMD simulations were then used to probe and compare the dynamics of both systems. Dynamic flexibility index (DFI) profiles derived from the simulations (right) reveal distinct differences in mobility between WT and ΔL11 ribosomes, with the ΔL11 mutant exhibiting increased mobility in functionally critical regions of the large (50S) subunit and decreased mobility at the dimerization interface of the small (30S) subunit.

In this study, we employed the workflow illustrated in Fig. 1 to analyze large-scale ribosome dynamics using coarse-grained molecular dynamics (CGMD) simulations. The atomic structure of the 70S ribosome was coarse-grained with the Martini 3 force field^34,35^ to generate models of both the wild-type (WT) ribosome and a mutant lacking the L11 protein (ΔL11). CGMD simulations were then performed to probe and compare the conformational dynamics of the two systems. We used the Dynamic Flexibility Index (DFI)^37–39^, which quantifies the relative flexibility per amino- or nucleic acid residue in response to perturbations of uniformly distributed random force. DFI profiles derived from the simulations highlight notable differences in residue-level flexibility between WT and ΔL11 ribosomes, with the mutant showing enhanced mobility in several functionally important regions of the large subunit (LSU) and reduced flexibility at the dimerization interface of the small subunit (SSU).

Our CGMD results indicate that removal of ribosomal protein L11 induces broad changes in the collective motions and dynamic coupling of the 70S ribosome. Deletion of L11 significantly reshapes mobility patterns across both subunits. We see generally increased mobility across the LSU and decreased at the binding interface of the SSU. Regions central to ribosomal activity—including the L1 stalk, L7/12 stalk, and the peptidyl transferase center (PTC)—exhibit elevated fluctuations, while other areas, such as the dimerization interface of the 30S subunit, become more constrained.

**Fig. 2:**
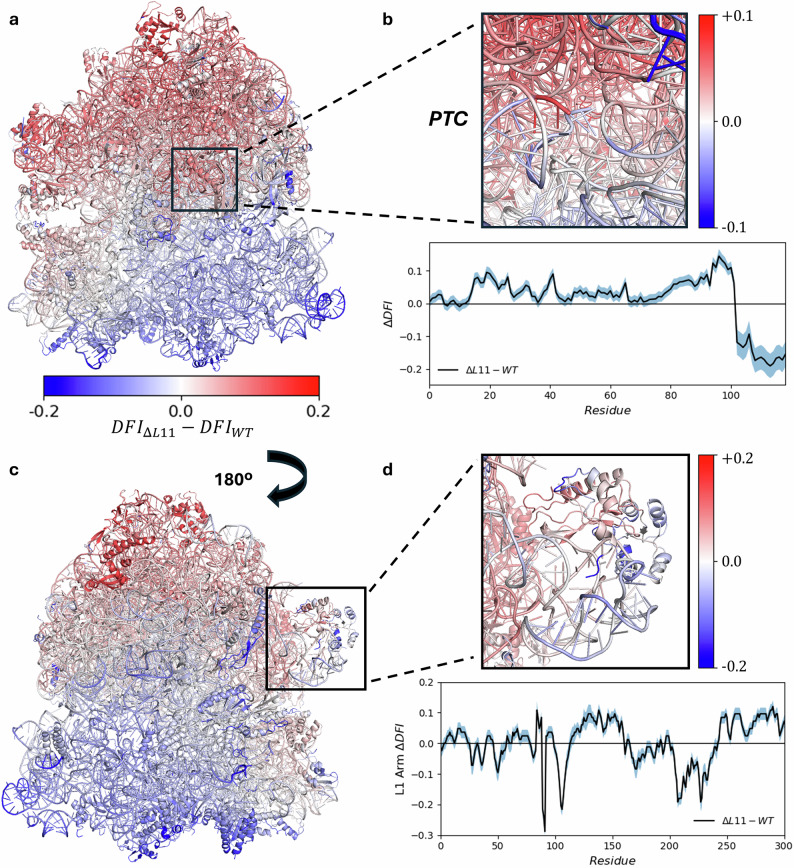
L11 deletion affects the dynamics of the translation apparatus. **a** Change in mobility, shown as the difference in the dynamic flexibility index (DFI) between ΔL11 and WT variants (front view). **b** The black square highlights the peptidyl transferase center (PTC), viewed from the front. In the ΔL11 variant, the average DFI of the PTC increases by approximately 10%, suggesting enhanced mobility. The graph shows the change in DFI of the PTC upon L11 deletion. Shaded band indicates mean ± s.e.m (*n* = 600). **c** Change in mobility between ΔL11 and WT variants (back view). **d** The black square highlights the location of the L1 RNA arm—ribosomal component pivotal for directing tRNA translocation. The base of the stalk exhibits increased mobility, whereas some hinges in the outer RNA part become more rigid. The graph shows the change in L1 arm DFI upon L11 deletion. Shaded band indicates mean ± s.e.m (*n* = 600).

These findings suggest a dual effect of L11 deletion: it increases fluctuations in the PTC that normally require tight coordination during translation^40^, while simultaneously restricting motions in regions that contribute to stress-induced structural transitions such as ribosome hibernation. Figure 2 quantifies these changes, showing that the ΔL11 ribosome displays approximately a 10% increase in flexibility at the PTC (Fig. 2b), a site central to peptide bond formation. To study the dynamics of PTC, we selected a sphere with a ~20 Å diameter around the center of geometry of residues U2506 and U2586, which resulted in approximately 120 residues in total. This alteration may compromise the catalytic precision needed for efficient translation, consistent with earlier studies demonstrating that perturbations near the PTC impair elongation and peptidyl transferase activity^41^. The dynamics of the L1 RNA arm, a mobile region involved in tRNA translocation^42^, are also modulated by L11 deletion. The base of the stalk exhibits increased mobility, whereas some hinges in the distal RNA region become more rigid (Fig. 2d). While the role of L11 in translation and its impact on canonical translation factors (e.g., EF-Tu, EF-G) has been well established in prior genetic, biochemical, and structural studies, including effects on elongation dynamics and translation efficiency (e.g., Van Dyke et al^43^), our results extend these findings by showing that L11 deletion disrupts coordinated domain motions within the ribosome. This adds an additional mechanistic explanation for the reduced translational efficiency observed in ΔL11 strains.

### Rigidification of the small subunit and HPF binding interface

**Fig. 3:**
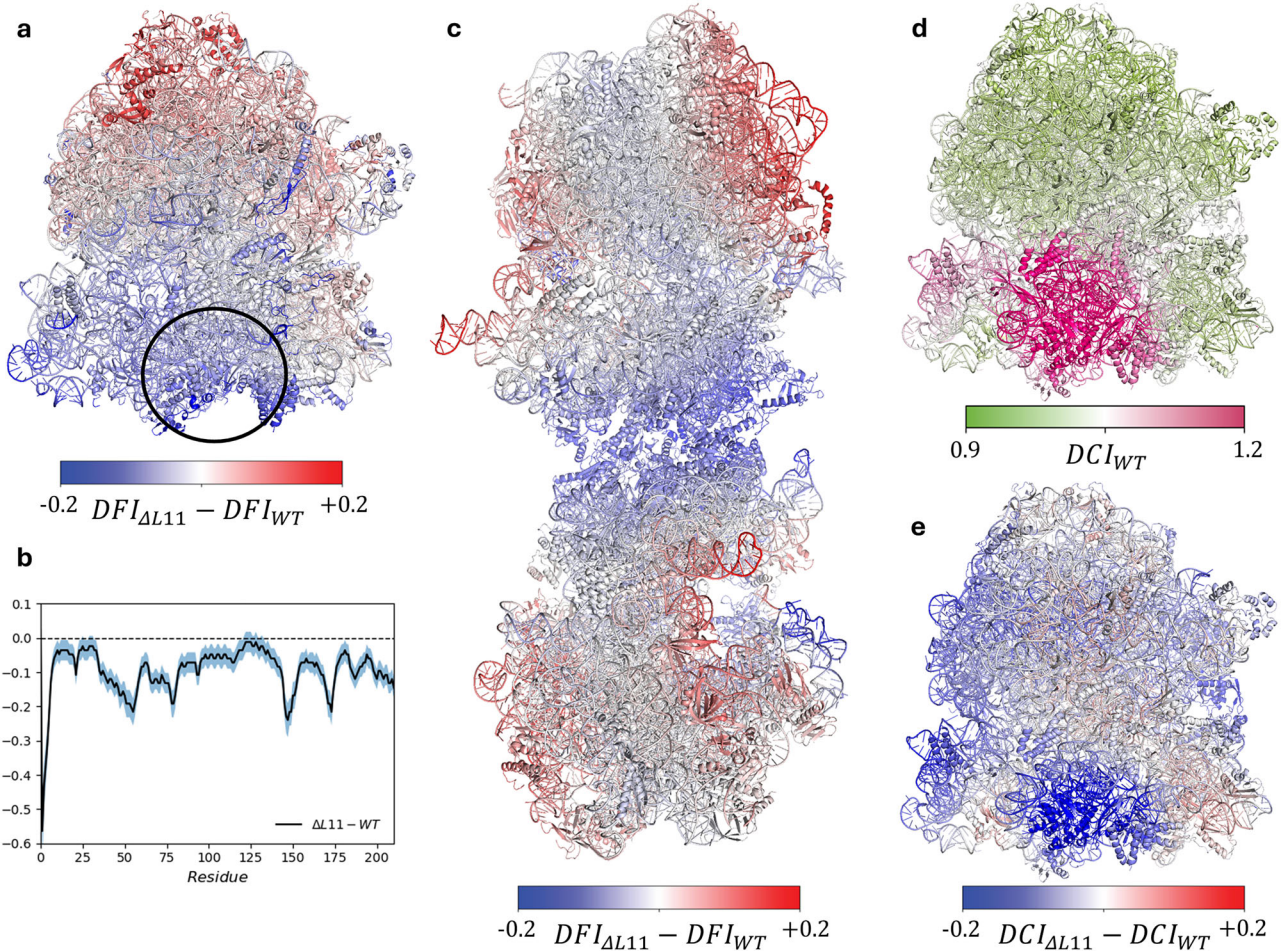
Rigidification of the small subunit. **a** Change in mobility, shown as the difference in dynamic flexibility index (DFI) between ΔL11 and WT variants. A notable decrease in mobility at the binding interface is observed. **b** The graph shows the change in DFI of the region around the HPF binding site upon L11 deletion. Shaded band indicates mean ± s.e.m (*n* = 600). **c** Difference in mobility between the ΔL11 and WT 100S ribosome dimers calculated from the elastic network model. **d** Dynamic coupling index (DCI) between the binding interface and the rest of the ribosome, and **e** The corresponding change upon L11 deletion. The ΔL11 ribosome shows weakened coupling between the binding site and its immediate surroundings, suggesting altered dimerization dynamics.

Our analysis shows that deletion of ribosomal protein L11 leads to pronounced rigidification at the HPF-binding interface of the SSU (Fig. 3). This increased rigidity also extends into the region that contributes to 100S dimer formation. The resulting decrease in dynamical fluctuations—and consequently in vibrational entropy—is predicted to favor 100S dimer association and thereby enhance dimer thermodynamic stability^44–46^. Importantly, panel B demonstrates that this enhanced rigidity persists in the dimeric state, as captured by our ENM-based calculations^47^. The rigidification of the dimer interface observed upon L11 deletion may enhance the stability of the dimer, as a reduction in flexibility typically correlates with an increase in binding affinity, as demonstrated in several other systems^48–50^. This suggests that the ΔL11 ribosome may potentially exhibit a delayed exit from dormancy due to a compromised ability to transition out of the hibernation state.

To further investigate the role of L11 as an allosteric inducer, we looked at the dynamic coupling index (DCI)^48,51,52^ profiles of the WT and ΔL11 variants. Similarly to DFI, DCI quantifies the relative coupling of residue *i* to residue *j* when residue *j* is perturbed. If residue *i* fluctuates a lot when residue *j* is perturbed, we say that residue *i* is highly coupled to residue *j*, as indicated by a high DCI value, with the average DCI across all perturbed residues being equal to 1. DCI analysis provides further support by showing that long-range communication between the HPF binding site and the broader ribosome network is markedly weakened upon L11 deletion. Because HPF-driven dimerization represents a central pathway for bacterial survival under stress conditions^18,19^, this decoupling underscores L11’s role not only in maintaining canonical translation but also in enabling adaptive transitions into hibernation states. While further experimental studies are required to determine if this translates to a delayed exit from dormancy in vivo, these dynamic shifts point toward a possible role for L11 in modulating the stability of dormant ribosomal states, acting as a structural rheostat for translation and stress response pathways.

These computational findings align with structural data from cryo-EM studies, which revealed that HPF binds in proximity to protein uS2 and rRNA helix h26, forming stable CTD–CTD contacts critical for establishing the 100S interface^18^. Within this context, we hypothesize that L11 may contribute to allosteric stabilization of global collective modes involved in this conformational adaptation. In ΔL11 ribosomes, loss of this support manifests as both rigidification and reduced coupling, changes that may account for the degradation of S2 and S3 proteins previously reported in HPF-deficient mutants^53,54^, as these proteins are left unprotected when the hibernation mechanism fails.

### Loss of coupling at the factor bingding cite and relA interface

**Fig. 4:**
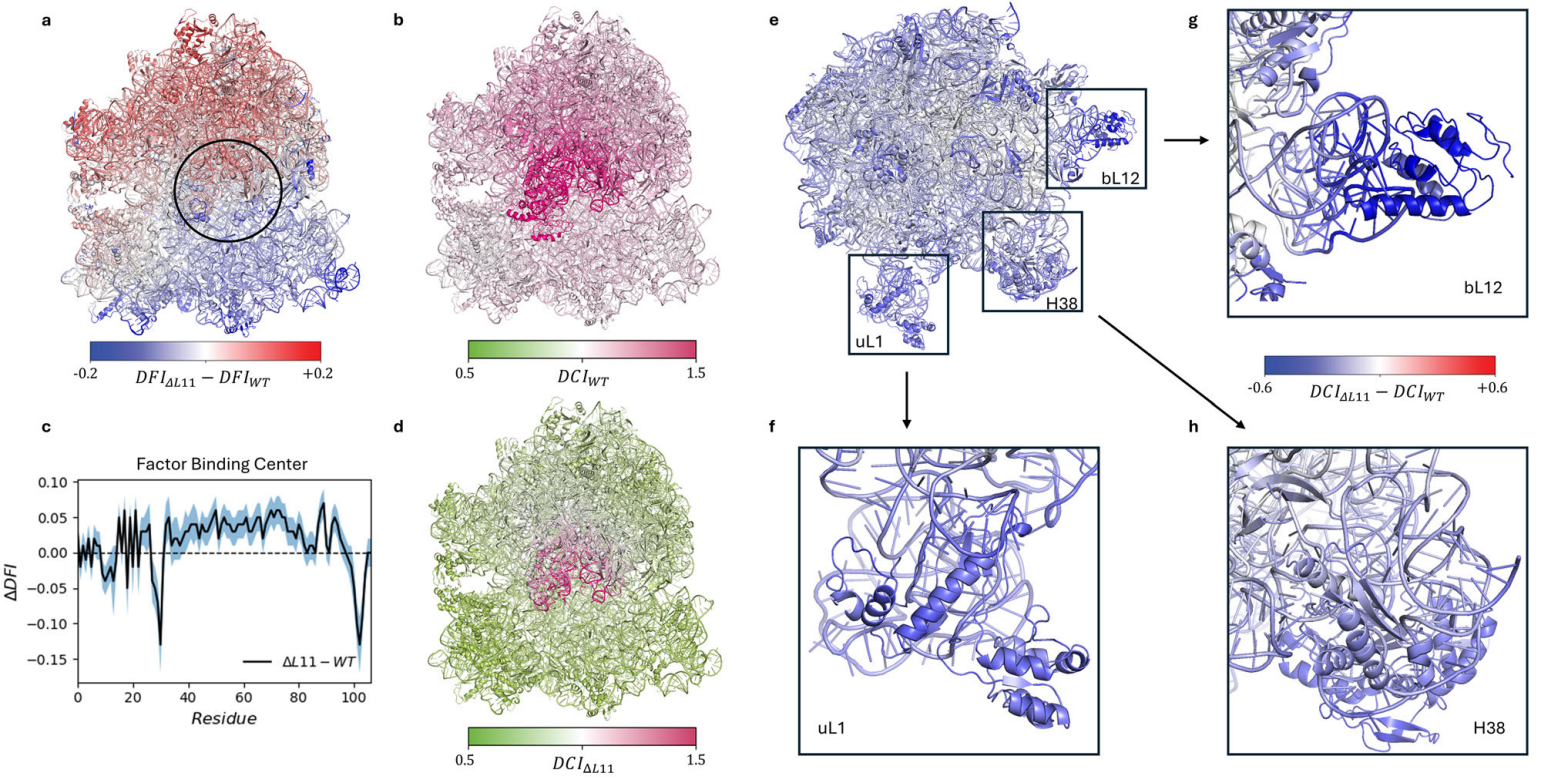
Effect of L11 deletion on FBC. **a** Change in mobility between the ΔL11 and WT ribosome variants, the black circle highlights the FBC. **b** Difference in dynamic flexibility index (DFI) of the region surrounding the FBC between the ΔL11 and WT variants. Shaded band indicates mean ± s.e.m (*n* = 600). **c** Dynamic coupling index (DCI) between the binding interface and the rest of the ribosome for the WT variant and **d** ΔL11 variant. The ΔL11 ribosome shows a significant loss of coupling between the binding site and the rest of the ribosome compared to the WT. **e** Change in DCI between the FBC and the regions of LSU upon deletion of L11. **f–h** Close-up views of the regions boxed in panel E (uL1, bL12 and H38).

**Fig. 5:**
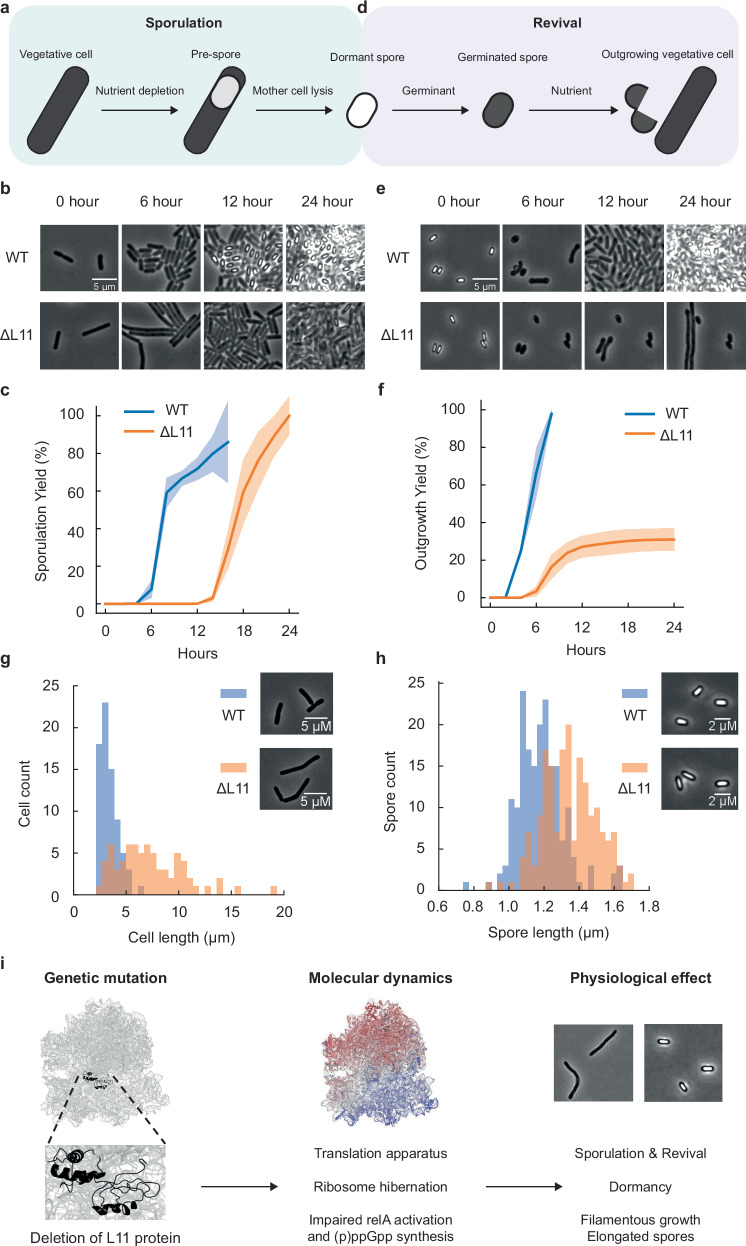
Physiological consequences of ΔL11 in sporulation, revival, and morphology. **a** Schematic view of the sporulation process. Under phase-contrast microscopy, the developing pre-spore becomes phase-bright within the phase-dark mother cell. **b** Phase-contrast microscopy filmstrips for representative WT and ΔL11 cells undergoing sporulation. Snapshots at 6-hour intervals after cells were applied to an agarose pad composed of RM medium for inducing sporulation. **c** Sporulation yield, defined as spores formed per vegetative cell present at the time of sporulation induction and scaled to the maximum observed value across all conditions. Data were analyzed at 2-hour intervals (mean ± 95% confidence interval; *n* = 6 per strain). Because vegetative cells continue to replicate after induction, yields may exceed one spore per initial vegetative cell. **d** Schematic view of the spore revival process. Spore phase-darkens during germination, followed by rupture of the spore coat and emergence of the outgrowing vegetative cell. **e** Phase-contrast microscopy filmstrips for representative WT and ΔL11 spores undergoing outgrowth. Snapshots at 6-hour intervals. **f** Outgrowth yield, expressed as the percentage of outgrowing spores, was analyzed at 2-hour intervals (mean ± 95% confidence interval; *n* = 6 per strain). **g** Histogram of WT and ΔL11 cell length (*n* = 75 cells per strain). Snapshots of representative cells are included on the right. **h** Histogram of WT and ΔL11 spore length (*n* = 73 spores per strain). Snapshots of representative spores are included on the right. **i** Summary schematic of L11 ribosomal protein deletion. Loss of L11 is predicted to disrupt the translational apparatus, hibernation state and stringent response, leading to defects in entry and exit from dormancy and cell and spore morphology.

As shown in Fig. 4b–d, the GTPase-associated center, also known as the factor binding center (FBC)^55–57^, exhibits a dramatic loss of coupling to the ribosomal core upon L11 deletion. FBC acts as the primary docking station and molecular switch for the various GTPases that drive the translation cycle forward. One such GTPase, ObgE^31^, plays a vital role in the onset of sporulation and the bacterial stringent response; thus the decreased dynamic coupling here may contribute to the impaired sporulation phenotypes observed in ΔL11 strain (Fig. 5). Feng et. al^33^ previously described how GTPase-ribosome interactions induce conformational changes to propagate allosteric signals across multiple domains and explicitly noted that the potential role of the FBC in activating the ObgE GTPase remained an open question. Consistent with this, Fig. 4c reveals a substantial reduction in coupling between the FBC and distal regions, indicating disruption of long-range communication pathways upon L11 deletion. To ensure that this effect is not specific to the assembled 70S ribosome, we additionally analyzed the isolated 50S subunit and observed a qualitatively similar loss of dynamic coupling (Fig. S1). Ultimately, disruptions in these pathways, whether through the loss of ribosomal proteins or post-translational modifications, can significantly alter cell fate decisions under stress^58^.

Another factor that binds to the ribosome and plays an important role in sporulation is RelA. RelA detects nutrient deprivation and catalyzes the production of (p)ppGpp, which signals the cell to initiate the stringent response—a pathway essential for the onset of sporulation in *B. subtilis*^59,60^. Extended analysis of the RelA binding region (Fig. S2) further illustrates the allosteric consequences of L11 deletion. In wild-type simulations, DCI values reveal robust coupling between the RelA binding pocket and the ribosomal core. This long-range dynamic linkage is lost in the ΔL11 variant, offering a structural rationale for the failure of these cells to initiate the stringent response under nutrient stress. These findings are in line with earlier biochemical studies indicating that L11 is required for (p)ppGpp synthesis via RelA activation^17,61^ and suggest that conserved allosteric signaling pathways rely on subtle modulations in subunit dynamics that can be easily disrupted by loss of hub proteins like L11. Taken together, these findings potentially provide a mechanistic rationale for the impaired sporulation in the ΔL11 variant.

### Experimental part. Sporulation defects in ΔL11

Our modeling results suggest that deletion of L11 should have an impact on the entry and exit from a dormant state. This expectation is specifically based on the altered interactions of the L11 ribosome with key regulators of dormancy as well as the disruption of the ribosome’s dimerization interface. Therefore, we investigated whether entry and exit into the most dormant state, spores, are impacted by the L11 deletion. We thus performed time-lapse microscopy to monitor and quantitatively determine the influence of L11 deletion on sporulation (entry into the spore state) as well as outgrowth (exit from the dormant state). Observation under phase-contrast microscopy revealed that deletion of L11 in *B. subtilis* results in a pronounced delay in sporulation initiation. Using the resuspension method (RM)^62^ to induce sporulation, the formation of phase-bright pre-spores within phase-dark mother cells was monitored over time (Fig. 5b). Filmstrip images show sporulation onsets between 6 and 12 hours in WT strain, while ΔL11 strain does not show spore formation until 12 to 18 hours post-induction (Fig. 5b). Quantitative analysis of spore formation further confirms this delay in sporulation in ΔL11 strain compared to WT (Fig. 5c). These phenotypes align with molecular dynamic modeling, which predicts that L11 deletion disrupts ribosome-Obg and ribosome-RelA interactions that are crucial for initiating sporulation, and reinforces that L11 is essential for proper entry into dormancy.

### Spore revival defects in ΔL11

Upon nutrient reintroduction, ΔL11 spores displayed a pronounced deficiency in revival capacity. Germination and outgrowth were initiated using L-alanine (as a germinant) and glucose (as a carbon source for growth) and tracked by changes in phase-contrast microscopy. Germinating spores phase-darken as the core hydrates, and outgrowing vegetative cells emerge through rupturing spore coats (Fig. 5d). Filmstrip images shows that WT spores begin outgrowth between 0 and 6 hours, while ΔL11 spores begin outgrowth between 6 and 12 hours, or fail to outgrow at all in the period of 24 hours (Fig. 5e). Quantitative analysis highlights this delay in outgrowth onset as well as reduction in the percentage of outgrowing spores in ΔL11 strain (Fig. 5f). These deficiencies likely stem from compromised translation apparatus and altered ribosome hibernation state, as predicted by modeling. In addition, defects in sporulation could further compromise the spore quality and revival potential. We also note that even though our experimental results are consistent with our modeling predictions, they do not exclude other possible factors. Regardless, these findings suggest a critical role for L11 in ribosome-mediated physiological transitions between dormant and active states in *B. subtilis* and highlight its importance for both entry and exit from dormancy.

### Morphology defects in ΔL11

We next examined cell morphology as another systems-level indicator of altered cellular regulation by the L11-deleted ribosome and its connection to stress-induced signaling. Consistent with prior studies linking L11 mutation to impaired RelA activation and reduced (p)ppGpp synthesis^63,64^, ΔL11 cells display pronounced elongation (Fig. 5g). This phenotype is similar to the known effects of (p)ppGpp depletion on cell septation^65^ and supports a link between L11 perturbation and altered ribosomal stress-response signaling. Notably, this elongated cell morphology also persists in the spore form: ΔL11 spores are longer in length compared to WT spores (Fig. 5h), suggesting that the underlying defect in septation becomes structurally embedded during sporulation. The elongated spores represent a distinct phenotypic effect of L11 deletion, consistent with its potential role in allosteric signaling.

### Functional consequences: sporulation and revival defects in ΔL11 strains

Consistent with the above structural insights, ΔL11 *B. subtilis* cells exhibit profound phenotypic abnormalities (Fig. 5i). Our experimental assays show delayed initiation of sporulation, delayed outgrowth, and decreased outgrowth fraction. These effects are qualitatively consistent with the reduced 100S dimerization propensity and altered coupling at hibernation, GTPase and RelA interfaces suggested by the simulations. Notably, the impact of L11 deletion on sporulation appears to differ from its effect on spore revival (outgrowth). While the timing delay between WT and ΔL11 is comparable for sporulation and outgrowth (approximately 6 hours), the efficiencies differ substantially: sporulation yield is nearly identical between WT and ΔL11, whereas spore revival efficiency in ΔL11 is only about one third of WT (Fig. 5f). These differences are also distinct from the overall growth-rate changes observed during vegetative growth. Many ΔL11 spores remain in a non-growing, non-dividing state for more than 24 hours after germination. This prolonged post-germination dormancy suggests a deeper failure to re-activate the regulatory programs required for re-entry into growth. Together, this behavior is not fully explained by a simple global reduction in translation efficiency and raises the possibility of process-specific perturbations associated with altered long-range ribosomal dynamic coupling.

Sporulation and revival are tightly linked to translational dormancy and ribosome integrity. Previous studies have established that long-form HPF proteins, such as those in *B. subtilis*, induce constitutive 100S formation and provide resistance against RNase degradation in dormant cells^18,66,67^. Our data suggest that L11 supports this system by preserving the structural plasticity and dynamic coupling necessary for HPF-mediated transitions. The absence of L11 may compromise efficient ribosome hibernation, potentially increasing susceptibility to degradation and thereby affecting the energetic economy required for rapid reactivation.

## Discussion

Together, these results suggest that L11 contributes to the allosteric regulation of ribosome function. Our data indicate that L11 facilitates long-range communication between distant sites—including RelA, HPF, and the PTC—through pathways of allosteric dynamic coupling. Loss of L11 appears to disrupt these interactions, impacting both translational efficiency and the capacity for adaptive persistence. Importantly, our findings echo a broader theme across cellular systems: that rigidity and flexibility must be precisely balanced for macromolecular machines to operate efficiently and adaptively^68^. In this context, L11 serves as a node that tunes the ribosome’s mechanical landscape, supporting the transitions between active translation and protective dormancy.

Future work will be needed to dissect the specific downstream consequences of altered dynamic coupling in ΔL11 ribosomes—for instance, by probing interactions with elongation factors, rescue complexes, or RNA chaperones. Nevertheless, our study establishes a foundational mechanistic model for how a single ribosomal protein can orchestrate global functional shifts in a complex molecular machine and shape the survival strategies of a bacterium under stress.

Finally, we show an intriguing link between modeling at the atomic scale and experiments at the cell physiological scale. Specifically, our modeling identified potential impacts of L11 deletion on bacterial dormancy and morphology. Experimental evidence shows how L11 deletion impacts both entry and exit from the spore state, and both cell and spore morphology. While these results are not a direct validation of our modeling results, they demonstrate that molecular-scale studies of ribosome allostery can potentially inform experiments at the cellular scale that would otherwise not have been obvious.

Through this interdisciplinary approach, we uncovered an unexpected connection between the L11 ribosome subunit and not only the ability of cells to sporulate or germinate, but even the morphology of cells and spores. Delayed sporulation and outgrowth are consistent with the allosteric impact of L11 deletion revealed by our modeling. Notably, the distinct impacts of L11 deletion on sporulation and spore revival argue against a simple uniform reduction in translation efficiency and instead point to pathway-specific perturbations arising from altered dynamic coupling. In particular, the increased structural rigidity of 100 s ΔL11 ribosome dimers is consistent with a more stable dormant state, providing a structural basis that may explain the observed delays in revival. The elongated shape of ΔL11 cells and spores is unexpected and will require future work to elucidate. To date, no studies have identified ribosome mutations that can alter the shape of spores. The discovery of an altered spore morphology underscores the utility of integrating molecular modeling with phenotypic analysis, as these structural predictions provided the basis for identifying previously unrecognized consequences of L11 deletion.

## Methods

### MD simulations

*Bacillus subtilis* ribosome structure was derived from a cryo-EM model with a resolution of 3.1 Å (PDB: 6HA1)^69^. All ligands, including antibiotics and the stalled peptide, were removed from the initial structure. To complete the model, missing proteins L10 and L11 were incorporated by superimposing the large subunit of PDB: 3J9W^70^ onto the initial structure. We then replaced the non-canonical nucleotides found within the experimental model by their chemically closest canonical counterparts to remain consistent with the available AA-to-CG mapping routines for the CG force-field. Finally, the resulting composite structures were subjected to brief energy minimization in vacuum, with position restraints applied to all heavy atoms, to remove any steric clashes introduced during the assembly process.

The CG ribosome was then placed in a periodic rhombic dodecahedron box with a minimum distance of 1.25 nm from the box faces. The model was then solvated using Martini 3 water beads. The system was neutralized by replacing the CG water beads with beads representing hydrated potassium and chloride ions. The final simulation box contained about 150 thousand particles. The proteins in CG representation were modeled using the Go-Martini approach^71^, while the RNA was simulated with the Martini 3 RNA parameters^34^.

MD simulations were performed using GROMACS version 2023^72^. The prepared systems were first subjected to energy minimization for 10,000 steps using the steepest descent algorithm to eliminate any unfavorable contacts. Following minimization, the systems were equilibrated in explicit water at room temperature (300 K). The initial equilibration phase lasted 10 ns with a 10-fs integration time step and included position restraints on the RNA/protein backbone (force constant k = 500 kJ mol^−1^ nm^−2^) to stabilize the overall structure. This was succeeded by an additional equilibration period of 100 ns without any position restraints, during which the time step was increased to 20 fs. After equilibration, production runs were performed for a duration of 10 μs per replicate. To ensure statistical significance and robustness, simulations were carried out in twelve independent replicates, each initialized with different velocity distributions, resulting in a total of 240 μs of CGMD data. During the equilibration and production stage, the temperature was maintained constant at 300 K using a modified Berendsen thermostat with a *τ*_*T*_ coupling constant of 1 ps. The pressure was maintained at 1 bar (isotropic coupling) using a modified Berendsen barostat during the initial equilibration (*τ*_*P*_  =  4 ps, compressibility of 3.0 × 10^−4^ bar^−1^) and a Parrinello-Rahman barostat (*τ*_*P*_  =  12 ps, compressibility of 3.0 × 10^−4^ bar^−1^) for the second equilibration and all production runs. Electrostatic interactions were cut off at 1.1 nm using the reaction-field method, and Lennard-Jones interactions were cut off at 1.1 nm using the potential-shift Verlet method.

### Dynamic flexibility index (DFI)

DFI provides a residue-level measure of structural flexibility by quantifying the extent to which each backbone position responds to perturbations propagated through the molecular network. The method combines Perturbation Response Scanning (PRS) with Linear Response Theory (LRT) to evaluate how local forces influence global dynamics. In PRS, residues are sequentially perturbed with a force F, resulting in displacements of all other residues, which are recorded as ΔR, the 3N×3N matrix of displacements. For an ENM, up to a proportionality factor, the response is given by:

1 $$\Delta R={H}^{-1}F,$$

where H is the 3N×3N Hessian matrix.

In MD simulations, for the case of a small constant force, the response can be calculated using LRT, yielding:

2 $$\Delta R={GF},$$

where G is the covariance matrix.

By applying unit isotropic perturbations sequentially to each residue, the displacement magnitude of residue j in response to a perturbation at residue i is given by:

3 $${R}_{{ij}}={\left|\left|\Delta {R}_{i}^{(j)}\right|\right|}$$

The DFI of residue *i* is then defined as:

4 $${DF}{I}_{i}=\frac{{\sum }_{j}{R}_{{ij}}}{\mathop{\sum }_{i}{\sum }_{j}{R}_{{ij}}}$$

A high DFI value indicates strong responsiveness (mobility), whereas a low value reflects structural rigidity. We then used the following procedure^73^ to assess convergence and errors: each trajectory was partitioned into windows of 200, 400, and 600 ns, each separated by a 200 ns lag, resulting in a total of *n* = 600 (12 replicas * 10,000 ns / 200 ns) windows across all trajectories. While slow drift can lead to statistical dependence between windows, the consistent result between different window sizes effectively captures the relevant motions on ~200–400 ns timescale while minimizing the impact of long-term drift. For each window, a covariance matrix was computed after aligning configurations to the first frame using only heavy backbone atoms. For our analysis, proteins were represented by their Cα atoms, while RNA was represented by the phosphate (P) backbone atoms. Average DFI profiles and their standard errors were then obtained across all windows and trajectories and compared across window sizes to assess consistency in the identification of flexible and rigid regions. Finally, non-overlapping 200-ns windows were selected for analysis.

### Dynamic coupling index (DCI)

DCI is an extension of DFI, developed to capture dynamic coupling between any given residue or a few residues and functionally critical residues, such as binding sites. This metric can locate sites that impact active site dynamics even at a distance through dynamic allosteric coupling. As defined, DCI is the ratio of the sum of the mean square fluctuation response of the residue *i* upon functional site perturbations (i.e., binding pocket) to the response of residue *i* upon perturbations on all residues:

5 $${DC}{I}\,_{i}^{j}=\frac{{R}_{{ij}}}{{\sum }_{j}{R}_{{ij}}/N}$$

Here, as earlier, ${R}_{{ij}}$ is the response fluctuation profile of residue *i* upon perturbation of residue *j*. Therefore, a higher DCI score of a residue with the functional sites would imply that perturbation at functionally important sites has a larger impact on that residue as compared to the rest of the molecule, indicating a higher coupling between the two. On the other hand, a lower DCI score would mean otherwise. Convergence and error estimation were performed following the same procedures as described in the DFI section.

### ENM models

To calculate the DFI and DCI of the 100S ribosome dimer (Fig. 3) and the ribosome–RelA complex (Fig. S1), we employed a coarse-grained representation based on ENMs. The ENM has been widely applied to proteins using a single atom per residue^47^ and to RNA structures using multiple atoms per nucleotide^74^. In this representation, the complexity of interactions among residues is reduced to node–node interactions. A node corresponds to a Cα atom for amino acids or a phosphorus atom for ribosomal nucleotides, and each node is connected to neighboring nodes via harmonic springs. For our analysis, proteins were represented by their Cα atoms, while RNA was represented by the phosphate (P) and ribose sugar (C1′) backbone atoms. All nodes were connected by harmonic springs with a distant-dependent spring constant. The spring force constant is inversely proportional to the sixth power of the pairwise distance, such that distant nodes contribute weakly to the interaction energy while nearby nodes exert stronger coupling. Metal ions co-crystallized with the ribosome, including magnesium, were also incorporated into the network by representing them as additional nodes connected to surrounding residues with springs of the same type. Within this framework, the collective response of the ribosome to perturbations can be evaluated using PRS and LRT described above, enabling sampling of the vibrational landscape of the ribosome and associated complexes.

### Bacterial strains

All experiments were performed using the *B. subtilis* strain NCIB 3610. The wild-type strain and pER450 vector were kind gifts from W. Winkler^75^ (University of Maryland). ΔL11 strain is generated using the pER450-based integration vector (pER450-rplK::cm^R^), which was constructed by Gibson assembly to contain 1 kb regions upstream and downstream of the *rplK* gene as recombination arms flanking a chloramphenicol resistance cassette for selection. NEB Turbo Competent *E. coli* cells (New England Biolabs, Ipswich, MA, USA) were used for vector replication. The construct was sequence verified and chromosomally integrated using a standard one-step transformation procedure^76^. Luria Broth (LB) agar plates containing 5 mg/L chloramphenicol antibiotic were used to select transformants. Transformed strain was confirmed by sequencing.

### Growth conditions

For imaging sporulation and vegetative cell morphology, desired *B. subtilis* strains were streaked on a fresh LB agar plate (with 5 mg/L chloramphenicol when appropriate) a day before the experiment and incubated at 37 °C overnight. A single colony of the desired strain was used for inoculation of 2 mL 20% LB medium and grown at 37 °C with shaking for 3 hours. For imaging spore revival and spore morphology, spores were prepared according to the Resuspension Method^62^, known to promote sporulation in *B. subtilis* strain NCIB 3610. Briefly, a single *B. subtilis* colony was used to inoculate 5 mL of LB and incubated overnight in a shaking incubator at 37 °C (200 rpm). The culture was diluted in 20% (v/v) LB at OD600 0.1 and incubated at 37 °C with shaking until it reached OD600 0.6–0.8. The culture was then centrifuged at 4000 rpm for 10 min, resuspended in 1 volume of pre-warmed RM, and incubated for three days at 37 °C in a shaking incubator (200 rpm). Resuspension medium (RM) is composed per 1 L of: 46 µg FeCl2, 4.8 g MgSO4, 12.6 mg MnCl2, 535 mg NH4Cl, 106 mg Na2SO4, 68 mg KH2PO4, 96.5 mg NH4NO3, 219 mg CaCl2, 2 g monosodium L-glutamate. The resulting spore suspension was washed with distilled water three times and suspended in 1 volume of distilled water. The spore suspension was stored at 4 °C. 2.5 µl of cell culture or spore suspension was applied onto 2% agarose pads made with RM medium. The pads were covered, left to air dry for 1 hour at 30 °C, and flipped onto a coverslip-bottom WillCo dish for imaging. The pads contained 10 mM of glucose for revival experiments to support outgrowth. Germination was induced by a liquid drop of L-alanine on top of the pads, calculated to be 10 mM final concentration.

### Time-lapse microscopy and analysis

Sporulation and spore revival were monitored with phase-contrast microscopy using an Olympus IX83 inverted epifluorescence microscope with a motorized stage (ASI). Images were taken with an Orca-Flash 4.0 LT+ camera (Hamamatsu) and an X-Cite Turbo light source (Excelitas Technologies) under a 100X objective lens. Collected images were processed with ImageJ (National Institutes of Health, http://imagej.net/nih- image/). For sporulation analysis, the Trainable Weka segmentation plugin from FIJI (ImageJ) was used to detect spores from each phase-contrast image. The number of spores was normalized by the number of cells at the start of imaging. For spore revival analysis, a threshold was applied and analyzed to create ROI masks of every single spore/cell. Any masks over 600 pixels were identified as outgrowing cells. The number of outgrowing cells was normalized by the number of spores at the start of imaging. For cell length measurements, a single time point prior to spore formation was selected for analysis. Cell length was determined by manually drawing lines through the longitudinal midpoint of each cell. For spore length measurements, a single time point before germination addition was analyzed. A threshold was applied to generate ROI masks corresponding to individual spores, and the major-axis length of each spore was quantified based on an ellipse fitted to the mask.

### Reporting summary

Further information on research design is available in the Nature Portfolio Reporting Summary linked to this article.

## Supplementary information

Supplementary Information

Description of Additional Supplementary Files

Supplementary Dataset 1

Supplementary Dataset 2

Supplementary Dataset 3

Reporting Summary

Transparent Peer Review file

## Source data

Source Data

## Data Availability

All data needed to evaluate the conclusions in the paper are present in the paper, Supplementary Materials or at [https://github.com/DanYev/Ribosomes]. Source data are provided in the source data file. Additional data is available upon request. PDB codes used in the work: 6HA1 (Cryo-EM structure of a 70S Bacillus subtilis ribosome translating the ErmD leader peptide in complex with telithromycin), 3J9W (Cryo-EM structure of the Bacillus subtilis MifM-stalled ribosome complex). Source data are provided with this paper.

## Code availability

The code used in this work is available on GitHub [https://github.com/DanYev/reforge] and Zenodo [10.5281/zenodo.19207979].
