# Supplementary material for: Ribosomal allostery as a potential regulator of bacterial dormancy
Source: Nat Commun. 2026 Jun 12;17:7938. doi: 10.1038/s41467-026-74199-2 (PMC13448080; doi:10.1038/s41467-026-74199-2)
Supplement: Supplementary file 1 [file 41467_2026_74199_MOESM1_ESM.pdf]

Ribosomal Allostery as a Potential Regulator of Bacterial Dormancy

Supplementary Information

Danis Yangaliev [1-2], Eun Chae Moon[3], Gürol M. Süel[3] *and S. Banu Ozkan[1-2]*

1. Center for Biological Physics, Arizona State University, Tempe, AZ, USA
2. Department of Physics, Arizona State University, Tempe, AZ, USA
3. Department of Molecular Biology, School of Biological Sciences, University of California San Diego, La Jolla, CA 92093 USA

Supplementary Figures

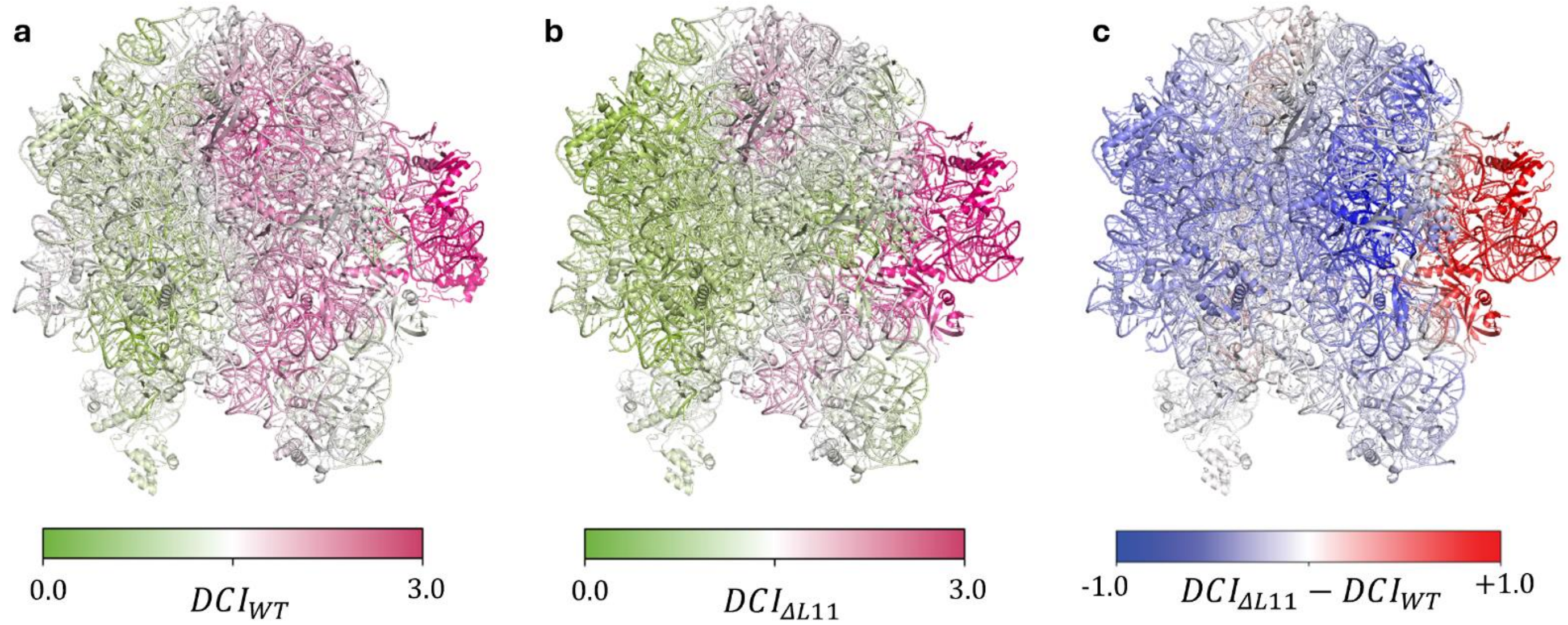

Fig. S1 *ENM analysis of the isolated 50S subunit in wild-type and ΔL11 systems*. (a) DCI between the factor-binding center (FBC) and the remainder of the 50S subunit in the wild-type, (b) the same analysis for ΔL11, and (c) the change in DCI upon L11 deletion, highlighting a loss of dynamic coupling within the large subunit.

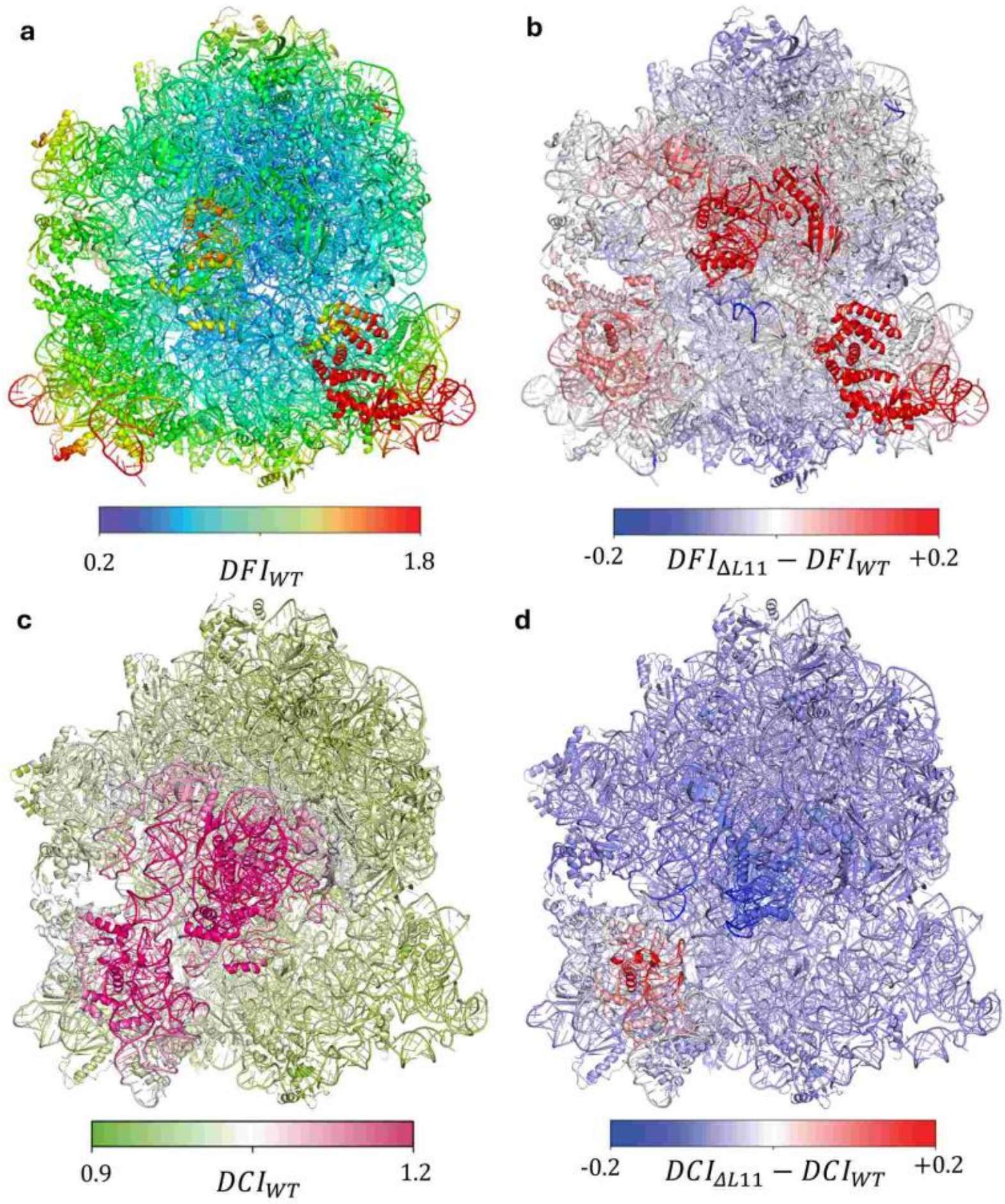

Fig. S2 *ENM model of the Ribosome+RelA complex*. **(a)** DFI profiles for the ribosome–RelA complex with L11 present and **(b)** the change in DFI upon deletion of L11. **(c)** DCI between the RelA binding interface and the ribosome and **(d)** the corresponding change upon L11 deletion. The ΔL11 variant shows weakened allosteric coupling between the RelA binding pocket and the ribosomal core. This disruption in long-range

communication provides a mechanistic rationale for the impaired ability of ΔL11 cells to activate the stringent response during nutrient stress.

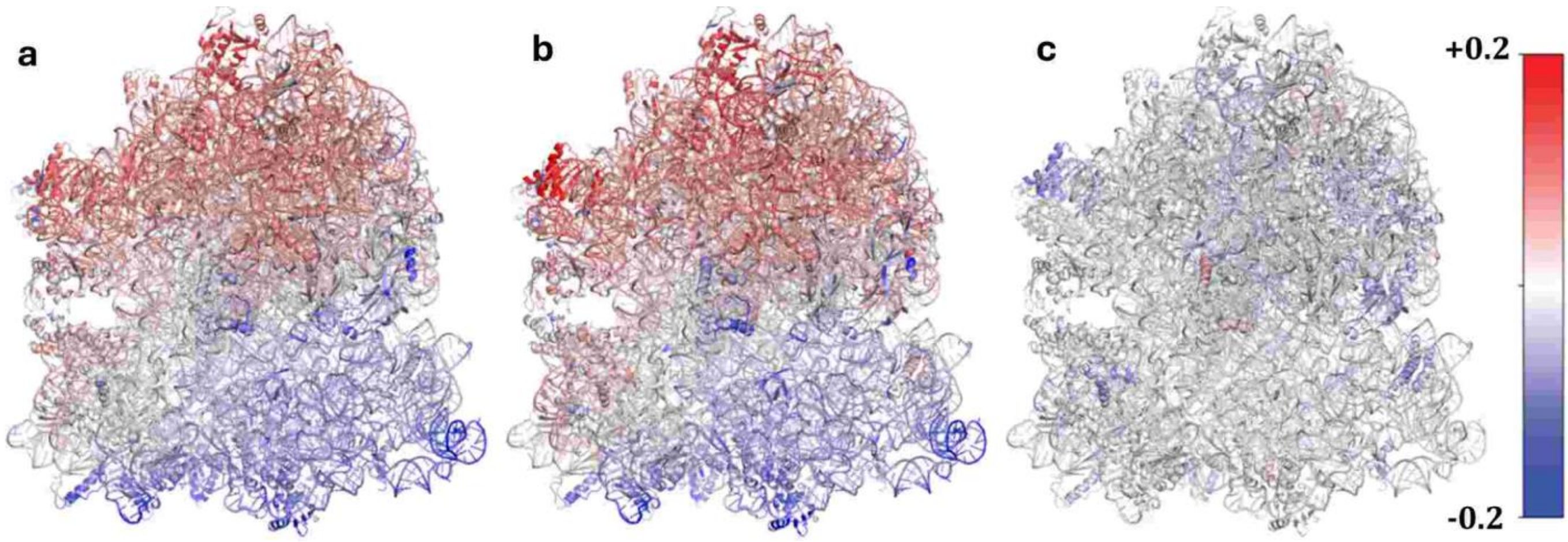

Fig. S3 *Robustness of ΔL11–WT DFI differences to alignment protocol.* ΔL11–WT differences in Dynamic Flexibility Index (DFI) computed using **(a)** whole-ribosome alignment, **(b)** separate alignment of the LSU and SSU, and **(c)** the difference between the two alignment approaches. While minor local deviations are observed, the overall spatial patterns and magnitudes of ΔL11–WT differences are preserved, indicating that the observed dynamical changes are not driven by alignment artifacts.

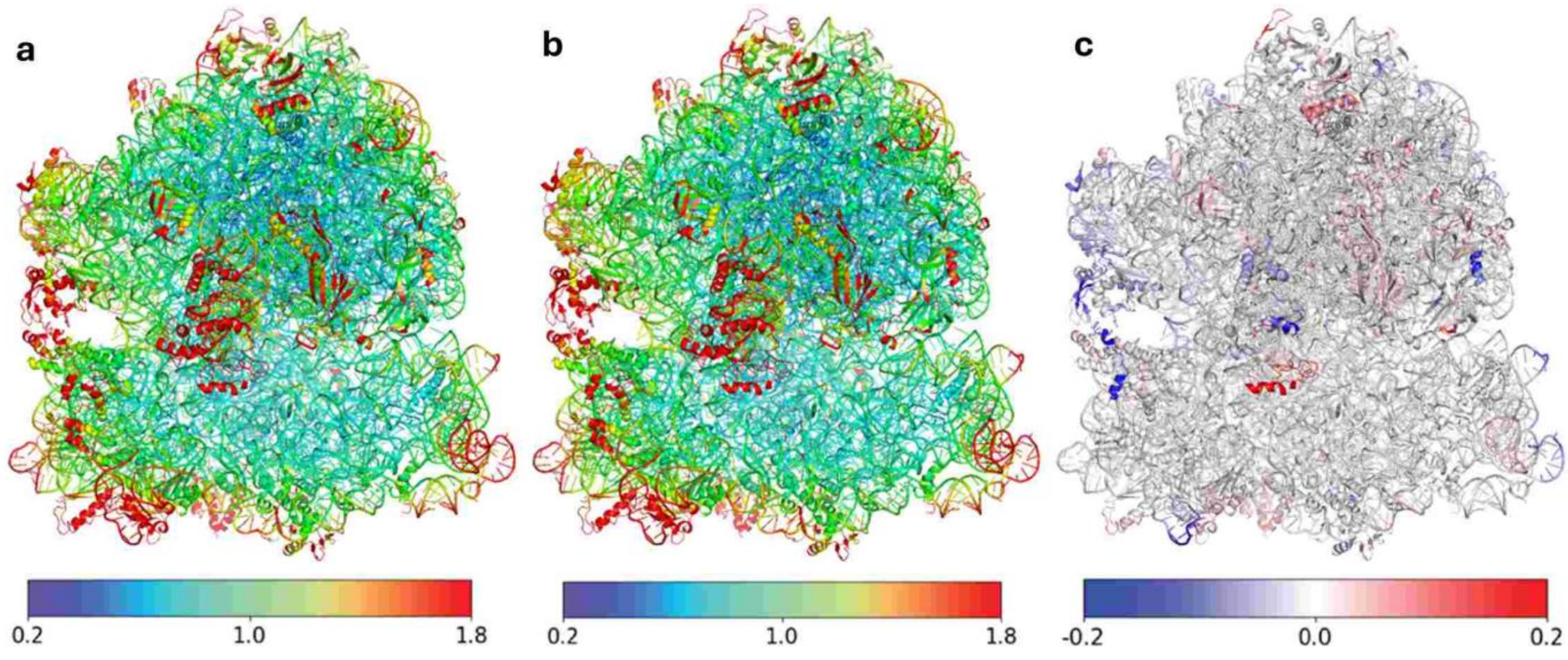

Fig. S4 *Robustness of convergence protocol.* We compare the DFI computed from non-overlapping 200-ns windows **(a)** with that obtained from 400-ns windows **(b)**, along with their difference **(c)**. While some deviations are observed in highly flexible peripheral regions, no differences occur in the ribosomal regions relevant to the analyses and conclusions presented in this work. Thus, the overlap in the longer windows does not affect the reported observations or lead to an underestimation of uncertainty.
