# Supplementary material for: Ribosomal allostery as a potential regulator of bacterial dormancy
Source: Nat Commun. 2026 Jun 12;17:7938. doi: 10.1038/s41467-026-74199-2 (PMC13448080; doi:10.1038/s41467-026-74199-2)
Supplement: Supplementary file 2 [file 41467_2026_74199_MOESM2_ESM.pdf]

Title: Supplementary Dataset 1

Description: data_colorcode_1.7z – archived ".pdb" files with data for the colorcoded figures

Title: Supplementary Dataset 2

Description: data_colorcode_2.7z – archived ".pse" files with data for the colorcoded figures

Title: Supplementary Dataset 3

Description: data_md.7z – md initial parameters and an example of the final structure
