# Supplementary material for: Ribosomal allostery as a potential regulator of bacterial dormancy
Source: Nat Commun. 2026 Jun 12;17:7938. doi: 10.1038/s41467-026-74199-2 (PMC13448080; doi:10.1038/s41467-026-74199-2)
Supplement: Supplementary file 4 [file 41467_2026_74199_MOESM7_ESM.pdf]

nature portfolio

Peer Review File

Ribosomal Allostery as a Potential Regulator of Bacterial Dormancy

Corresponding Author: Dr S. Banu Ozkan

This file contains all reviewer reports in order by version, followed by all author rebuttals in order by version.

Version 0:

Reviewer comments:

Reviewer #1

(Remarks to the Author)
This is an extremely important study of the effects of the ribosomal proteins L11on the dynamics and allostery of the ribosome, showing that is presence/absence controls hibernation. This is important because there the roles of ribosomal proteins have been unknown and this was a large open question. The word definitively shows how the presence or absence of this protein affects the interactions with other factors. This work is important in proving with simulations and experiments how L11 controls aspects of ribosome, essentially turning it on and off.
In the background you might mention the RNA World hypothesis that RNA preceded the emergence of proteins so the proteins obviously weren't important. We did a preliminary investigation of the dynamics of the ribosome, with and without the proteins, and showed that there was a strong similarity between their dynamics. You could add something to the introduction about this old point of discussion.

We investigated computationally the effects of removing proteins from the small subunit that showed some non-homogeous specific effects (Yan A, Wang Y, Kloczkowski A, Jernigan RL. Effects of protein subunits removal on the computed motions of partial 30S structures of the ribosome. J Chem Theory Comput. 2008 4:1757-1767. doi: 10.1021/ct800223g. PMID: 19771145; PMCID: PMC2665723.).
There are other studies that indicate some possible additional levels of control the also involve L11, by modifications to L11, such as methylation or binding to thiostrepton. These are important and should be mentioned, as well as being possible subjects for future study (Walukiewicz HE, Farris Y, Burnet MC, Feid SC, You Y, Kim H, Bank T, Christensen D, Payne SH, Wolfe AJ, Rao CV, Nakayasu ES. Regulation of bacterial stringent response by an evolutionarily conserved ribosomal protein L11 methylation. mBio. 2024 Oct 16;15(10):e0177324. doi: 10.1128/mbio.01773-24. Epub 2024 Aug 27. PMID: 39189746; PMCID: PMC11481523
Bailly C. The bacterial thiopeptide thiostrepton. An update of its mode of action, pharmacological properties and applications. Eur J Pharmacol. 2022 Jan 5;914:174661. doi: 10.1016/j.ejphar.2021.174661. Epub 2021 Dec 3. PMID: 34863996).

The present paper is clearly written and carefully explains all details of the simulations and experiments.

(Remarks on code availability)

Reviewer #2

(Remarks to the Author)
In their manuscript "Ribosomal Allostery as a Regulator of Bacterial Dormancy", Yangaliev et al. used coarse-grained molecular dynamics simulations to investigate the effect of the deletion of ribosomal protein L11. The authors attribute differences in fluctuations to allostery. Deciphering allostery on the ribosome is certainly an important and challenging topic.

However, I have a number of major methodological concerns, which suggest that the results may not sufficiently support the conclusions.

Major concerns:

(1) My main concern is that the fluctuation analysis might suffer from alignment artifacts rendering it difficult to judge if the observed effects are really due to intrinsic dynamics, or rather due to the chosen alignment. E.g., some of the presented results could potentially be explained by a combination of different intersubunit rotation dynamics and artifacts introduced by structure alignment. When aligning trajectories using atoms from both subunits, the LSU has more weight in the alignment (more atoms). The result of this alignment would be that SSU appears less mobile compared to the LSU. A difference between WT and ΔL11 in how pronounced intersubunit rotation is, might then lead to the observed differences in dynamics and would not require any allostery (Fig. 1).

A possible test might be to align the subunits separately and then perform the analysis.
Further, the reduced number of atoms in the LSU in the ΔL11 might noticeable perturb the alignment by increasing the weight of SSU atoms compared to the WT.

(2) As part of the Martini setup, the authors use an elastic network approach and then extract DFI and DCI which is based on linear response theory. Use of linear response is appropriate for small molecules, where the covariance matrix indeed relates to the Hessian as in eq (2). The free energy landscape of larger macromolecues and macromolecular complexes, however, has many local minima, which prevent straightforward application of linear response theory. Specifically, the covariance matrix contains all entropic contributions to the free energy, whereas the Hessian does not. As the DCI is derived from DFI, it may suffer from the same flaws. To what extent their results depend on the parameter choices, e.g. the elastic network model? How well is the coupling described by the linear response?

(3) The authors claim that “These findings suggest a dual effect of L11 deletion: it increases fluctuations in regions that normally require tight coordination during translation, while simultaneously restricting motions in regions that contribute to stress-induced structural transitions such as ribosome hibernation”. In my opinion this is an oversimplification since the decoding center is located on the SSU, a region with decreased fluctuations (according to Fig.1), and decoding requires a very tight coordination to monitor correct codon-anticodon base pairing.

(4) Also, the comparison to the experiments does not provide a convincing validation of the computational results, because the experiments do not probe fluctuations or allostery in the ribosome; rather, they are, if at all, rather indirectly related to the presented computational results. E.g., the observed difference in sporulation behaviour and the differences of observed binding site dynamics could potentially be due to many other effects. E.g., the ΔL11 variant might simply lead to slower translation?

(5) Assuming that changes in intersubunit rotation dynamics contribute markedly to the observed reduced SSU mobility, why should this affect dimerization? As the binding site does not involve the two subunits, I would think that the local flexibility of the binding site would have a larger effect on the binding than the dynamics of intersubunit rotation.

(6) In the Methods section, the authors write that the structure was completed to account for proteins missing in the experimental structure. How was that done?

(7) The authors have investigated the convergence of these observables by partitioning their trajectories into “overlapping windows”. Doesn’t this overlap compromise independence of the calculated observables and thereby lead to an underestimation of the s.e.m.? As the protocol of partitioning the data is not clearly described, I might be missing something, though.

(8) Is the coarse grained approach and the used force field accurate enough to support the conclusions? In a recent preprint, Cikhart et al. (https://arxiv.org/abs/2503.21357) tested the Martini (2.2) forcefield with several four different elastic networks and found substantial differences in the resulting dynamics. They also provided a direct comparison to all-atom MD simulations of the same length, showing that some elastic networks agreed with the all-atom simulations better than others. It would be instructive to compare the setup used here to the tested ones.

Minor concerns

(9) Several statements in the Introduction lack the supporting references:
“… naturally occurring ΔL11 variant”
“Ribosomal protein L11 occupies a particularly strategic position in this context: located at the base of the L7/L12 stalk, L11 is a key communication node that couples factor binding, GTPase activation, and structural transitions across the large subunit.“
“Classical biochemical studies have long implicated L11 in recruiting translation factors and in regulating stress responses, ..”.

(10) “preserving the global collective modes necessary for this conformational adaptation”
Are these collective modes known? Is there a comparison?

(11) “While these results are not a direct validation of our modeling results, they demonstrate that atomic scale studies of ribosome allostery […]” Coarse-grained simulations are not atomic scale.

(12) “This increased rigidity extends across the interface responsible for 100S dimer formation, accompanied by a reduction in local mobility. In particular, the ΔL11 ribosome shows a pronounced decrease in vibrational entropy” In the Methods it is not clarified how local mobility was computed. Is it vibrational entropy? How was the vibrational entropy computed?

(Remarks on code availability)

Reviewer #3

(Remarks to the Author)
In the manuscript „Ribosomal Allostery as a Regulator of Bacterial Dormancy” the authors apply ‘molecular dynamics simulations of wild-type and L11-deleted (ΔL11) ribosomes reveal that L11 functions as a global allosteric regulator coordinating communication between the ribosomal stalk and the peptidyl transferase center’.

Authors use sporulation in Bacillus subtilis as a model to test their working model. They discuss the role of L11 on the action of ribosomal hibernation factors HPF and RMF, proteins RelA and Obg.
It is surprising, the authors do not investigate what will happen to canonical translation factors such as EF-Tu or EF-G in the L11-deleted strain. Their function will be impaired, resulting in a strong phenotype.
Experiments presented in this manuscript do not support any of the claims of the authors and need to be redesigned. Some in vitro work would help support the model. When is L11 absent from the ribosome? What fractions of the ribosome (wild type) would lack L11?

This manuscript is far from being acceptable for publication.

(Remarks on code availability)
I am not an expert on molecular dynamics

Version 1:

Reviewer comments:

Reviewer #1

(Remarks to the Author)
The authors have responded in appropriate ways to all of the points made in the referees' reports.

(Remarks on code availability)

Reviewer #2

(Remarks to the Author)

The authors have markedly improved the manuscript and clarified several of my concerns. However, several other concerns remain that are not minor. Mainly the conclusions are still overstating the actually achieved confidence, the methods are in part not described to sufficient detail such that the study is reproducible, and the Introduction needs to more carefully reflect what is known and how this motivates the present hypothesis. I think these issues must and can be addressed. I’ll list my comments below, following the numbering of my initial review.

(1) Based on the results of the test, I now agree that the particular type of structure alignment used does not appear to critically affect the results.

(2) The authors have sufficiently addressed this point. I agree it was mostly a matter of unclear writing, which is now clarified both in the reply as well as in the manuscript.

(3) The revision sufficiently addresses my point.

(4) Here I remain unconvinced. The remaining lack of a direct validation of the coarse-grained simulations via comparison to experiments renders it hard to judge how reliable the results are. In the previous paper about the development of the coarse-grained model, the model was compared to atomistic MD simulations using fluctuations. How well simulations of this model (which is the basis for the DFI and DCI calculations) can predict long-range allostery is not clear to me and needs to be directly checked against experiments for at least one of the presented mechanisms or for a similar combination of model and

experiment. In my opinion, therefore, with the data at hand, the presentation of the conclusions is still too overconfident.

As an example, in the section "Rigidification of the small subunit and HPF binding interface", based on the reduction in fluctuation at the 100S dimer interface upon removal of L11 observed in the simulations and ENN calculations of single and dimerized ribosomes, the authors suggest that 100S formation is favored for the ΔL11 variant. Based on this suggestion, they suggest that "ΔL11 ribosome may exhibit a delayed exit from dormancy". Given the chain of uncertain inferences and the lack of discussion of alternative explanations, the final paragraph is an overstatement: "This observation further highlights the critical role of L11 in regulating the dynamic equilibrium between active and dormant ribosomal states, supporting its function as an allosteric inducer of translation and stress response pathways."

As a further example, in the final paragraph of the Discussion, the authors state that "the higher affinity of ΔL11 ribosomes to form 100s dimers is consistent with a more stable dormant state that can explain delayed revival.". However, the affinity was never measured or estimated, so stating the higher affinity as an established fact is not justified.

The authors need to adapt their wording of the conclusions to the actual strength or weakness of the evidence they provide.

(5) Here I disagree. While a full-scale intersubunit rotation is indeed not expected on the time scale of the simulations, in previous 100-ns long all-atom MD simulations (reference 10), rotations in the range of 5° were observed for several translocation intermediate states. Therefore, rotation in at least in this range would also be expected during the 200-ns long windows. I suggest the authors comment on this discrepancy or provide a reason for why this is not observed in their coarse-grained simulations.

(6) Thanks for the explanation, but for the study to be reproducible, the manuscript (Methods) needs to contain the exact protocol of how the structure was complemented. Further, just telling that "appropriate supplemental ribosomal structures" were used is insufficient. The used structure (PDB 6HA1) is a cryo-EM structure of a ribosome containing a stalled peptide and an antibiotic. Are the stalled peptide and the antibiotic part of the simulated model? How would that specific complex affect the results?

(7) The non-overlap of the windows is necessary but not sufficient for statistical independence. 200 ns is short compared to timescales of ribosomal motion (e.g. subunit rotation, see the above reply by the authors) and slow motions (a drift over longer timescales) could make the windows statistically dependent. The standard error of the mean would then underestimate the statical error (e.g. in Fig. 2b, d) and could lead to unsupported conclusions. The authors need to comment on this possibility.

In the Methods the authors then write "We then used the following procedure66 to assess convergence and errors: each trajectory was partitioned into windows of 200, 400, and 600 ns, each separated by a 200 ns lag, resulting in a total of n = 600 windows across all trajectories." And later "Finally, non-overlapping 200-ns windows were selected for analysis.", which should then be less than 600 windows. The Figure captions, however, say that n is 600. Which is correct?

(8) Thanks for the clarification.

(9) Regarding the new reference 27 (Dewachter, L. et al.), I don't see how this reference, which does not contain any structural information of the ribosome and does not even mention L11, supports the statement. Further, references 27 (Dewachter et al.) and 29 (Chakraborty et al.) are given as reference for the sentence "Furthermore, GTPase Obg, a master regulator of sporulation in B. subtilis, binds near the base of the L7/L12 stalk in close proximity to ribosomal protein L11.". From the references, it appears that Obg binds to the isolated large subunit and prevents ribosome assembly. How does the role of Obg to prevent assembly relate to the presented work where allostery in the assembled ribosome is studied? Overall, the introduction needs to be markedly clearer in presenting what is known about the role of L11 and how the previous results relate to the hypothesized allostery. Currently, the introduction does not convincingly motivate the hypothesis of L11 being involved in allostery.

The remaining minor comments have been sufficiently addressed.

One additional minor comment: The bacterial protein L11 corresponds to protein L12 in yeast and human ribosomes. To avoid confusion, I suggest to either switch to or at least mention the commonly used name uL11 (see https://doi.org/10.1016/j.sbi.2014.01.002 for the nomenclature).

(Remarks on code availability)

Reviewer #3

(Remarks to the Author)
I have some additional questions and requests for the authors.
How is sporulation efficiency calculated? Pictures provided for mutant cells do not look like nearly 100% spores.
While the results of the experiment presented are interesting and clearly show differences compared to the wild-type Bacillus strain, they are insufficient to support the statements in the manuscript.
What happens to the ribosomes in the mutant cells? The effect on hibernation and RNA degradation can be tested. Authors

should check ribosomes using sucrose gradient centrifugation and the integrity of rRNA by isolating the RNA and testing it on a gel or by gel capillary electrophoresis. Observed differences in sporulation efficiency and germination could be a secondary effect. The authors should show the direct link to ribosome hibernation and/or degradation.
Why are Bacillus cells not recovering after germination? Even after 24 hrs they are not dividing and they do not re-enter sporulation.

(Remarks on code availability)

Version 2:

Reviewer comments:

Reviewer #2

(Remarks to the Author)
The authors have now addressed all my comments satisfactorily.

(Remarks on code availability)

Reviewer #3

(Remarks to the Author)
The authors of the manuscript „Ribosomal Allostery as a Regulator of Bacterial Dormancy" did not provide convincing experimental data supporting their hypothesis.

Deletion of L11 ribosomal protein will affect the entire proteostasis of the sporulating Bacillus subtilis. The authors do not show any evidence directly pointing the loss of protein with dormancy of Bacillus. Impaired sporulation can be linked to defective translation, since deletion of L11 will also affect canonical translation factors such as EF-G and EF-Tu. It is known that perturbations to the ribosome such as deletion of ribosomal proteins, can affect maturation of spores. For example deletion of L31 protein as shown by Sinai et al PMID: 25661487 or paralogs of L31, L33 and S14 as shown by Iwanska et al PMID: 39169056 show defects to the spore maturation and dormancy. The authors of this manuscript are not showing what happens to the ribosomes. There is no in vitro monitoring of the translation (AHA, OPP PMID: 39753535, PMID: 39169056), no sucrose gradient polysome profiling (formation of 100S), monitoring of rRNA degradation (PMID: 22209493), no direct evidence. Translation in sporulation is fairly well described (PMID: 39753535, PMID: 39169056) and so is germination (PMID: 25661487). The authors do not show what happens to the ribosomes in their experiment, either in vivo or in vitro.

The experiments presented in this paper show the consequences of the impaired proteostasis of the cell. And we observe indirect consequences, secondary effects. To confirm the hypothesis, the authors should show direct evidence.

The authors claim that RelA action and ppGpp synthesis are affected. But they still observe spore formation. They should show that indeed ppGpp synthesis is abolished. Moreover, inhibition of ppGpp synthesis during sporulation should result in no spore phenotype (Wexselblatt et al PMID: 23028324), which is not the case in the presented work.

The elongated phenotype of L11 also does not explain the hypothesis. Again, they show no direct evidence, no localization of translation, and no septation (which could be done using a membrane stain).

The experimental part needs to be redesigned and redone.

(Remarks on code availability)

Open Access This Peer Review File is licensed under a Creative Commons Attribution 4.0 International License, which permits use, sharing, adaptation, distribution and reproduction in any medium or format, as long as you give appropriate credit to the original author(s) and the source, provide a link to the Creative Commons license, and indicate if changes were made.
In cases where reviewers are anonymous, credit should be given to 'Anonymous Referee' and the source.
The images or other third party material in this Peer Review File are included in the article's Creative Commons license, unless indicated otherwise in a credit line to the material. If material is not included in the article's Creative Commons license and your intended use is not permitted by statutory regulation or exceeds the permitted use, you will need to obtain permission directly from the copyright holder.
To view a copy of this license, visit https://creativecommons.org/licenses/by/4.0/

REVIEWER COMMENTS

Reviewer #1 (Remarks to the Author):

This is an extremely important study of the effects of the ribosomal proteins L11on the dynamics and allostery of the ribosome, showing that is presence/absence controls hibernation. This is important because there the roles of ribosomal proteins have been unknown and this was a large open question. The word definitively shows how the presence or absence of this protein affects the interactions with other factors. This work is important in proving with simulations and experiments how L11 controls aspects of ribosome, essentially turning it on and off.
In the background you might mention the RNA World hypothesis that RNA preceded the emergence of proteins so the proteins obviously weren't important. We did a preliminary investigation of the dynamics of the ribosome, with and without the proteins, and showed that there was a strong similarity between their dynamics. You could add something to the introduction about this old point of discussion.

We investigated computationally the effects of removing proteins from the small subunit that showed some non-homogeous specific effects (Yan A, Wang Y, Kloczkowski A, Jernigan RL. Effects of protein subunits removal on the computed motions of partial 30S structures of the ribosome. J Chem Theory Comput. 2008 4:1757-1767. doi: 10.1021/ct800223g. PMID: 19771145; PMCID: PMC2665723.).
There are other studies that indicate some possible additional levels of control the also involve L11, by modifications to L11, such as methylation or binding to thiostrepton. These are important and should be mentioned, as well as being possible subjects for future study (Walukiewicz HE, Farris Y, Burnet MC, Feid SC, You Y, Kim H, Bank T, Christensen D, Payne SH, Wolfe AJ, Rao CV, Nakayasu ES. Regulation of bacterial stringent response by an evolutionarily conserved ribosomal protein L11 methylation. mBio. 2024 Oct 16;15(10):e0177324. doi: 10.1128/mbio.01773-24. Epub 2024 Aug 27. PMID: 39189746; PMCID: PMC11481523
Bailly C. The bacterial thiopeptide thiostrepton. An update of its mode of action, pharmacological properties and applications. Eur J Pharmacol. 2022 Jan 5;914:174661. doi: 10.1016/j.ejphar.2021.174661. Epub 2021 Dec 3. PMID: 34863996).
The present paper is clearly written and carefully explains all details of the simulations and experiments.

We thank the reviewer for these insightful comments. We have incorporated the relevant references accordingly.

Reviewer #2 (Remarks to the Author):

In their manuscript “Ribosomal Allostery as a Regulator of Bacterial Dormancy”, Yangaliev et al. used coarse-grained molecular dynamics simulations to investigate the effect of the deletion of ribosomal protein L11. The authors attribute differences in fluctuations to allostery. Deciphering allostery on the ribosome is certainly an important and challenging topic.

However, I have a number of major methodological concerns, which suggest that the results may not sufficiently support the conclusions.

Major concerns:

(1) My main concern is that the fluctuation analysis might suffer from alignment artifacts rendering it difficult to judge if the observed effects are really due to intrinsic dynamics, or rather due to the chosen alignment. E.g., some of the presented results could potentially be explained by a combination of different intersubunit rotation dynamics and artifacts introduced by structure alignment. When aligning trajectories using atoms from both subunits, the LSU has more weight in the alignment (more atoms). The result of this alignment would be that SSU appears less mobile compared to the LSU. A difference between WT and ΔL11 in how pronounced intersubunit rotation is, might then lead to the observed differences in dynamics and would not require any allostery (Fig. 1).

A possible test might be to align the subunits separately and then perform the analysis. Further, the reduced number of atoms in the LSU in the ΔL11 might noticeable perturb the alignment by increasing the weight of SSU atoms compared to the WT.

We thank the reviewer for raising this point. To directly address this concern, we performed an additional control analysis in which the LSU and SSU were aligned *separately,* rather than using a single whole-ribosome alignment as in the original analysis. The resulting ΔL11–WT DFI difference maps are shown in the new supplemental figure S2, which compares three cases:(a) the original whole-ribosome alignment (as in Fig. 2A), (b) the reviewer-suggested separate LSU/SSU alignment, and(c) the difference between these two alignment strategies.

While minor local deviations are observed, the overall spatial patterns and magnitudes of the ΔL11–WT differences remain highly consistent across alignment protocols. Importantly, the differences between alignment schemes (panel c) are substantially smaller than the ΔL11–WT signal itself, indicating that the conclusions of this study are not sensitive to the choice of alignment.

We further note that both WT and ΔL11 trajectories were subjected to identical alignment procedures, and therefore any systematic alignment bias would affect both

systems similarly. While the reviewer correctly points out that the LSU contains more atoms and therefore carries greater weight in a whole-ribosome alignment, the quantitative impact of L11 removal on this weighting is minimal. Specifically, the L11 backbone corresponds to 141 coarse-grained beads out of approximately 11,000 beads in the full ribosome, implying a maximal alignment-weighting difference on the order of ~1.3%. In contrast, the dynamical differences observed in our DFI analysis are on the order of ~10%, i.e., nearly an order of magnitude larger.

Thus, while we agree that alignment choices can, in principle, influence apparent fluctuations, the magnitude and robustness of the observed ΔL11–WT differences cannot be explained by alignment artifacts or intersubunit rotation effects alone. Instead, the results support a genuine redistribution of intrinsic ribosomal dynamics upon L11 deletion.

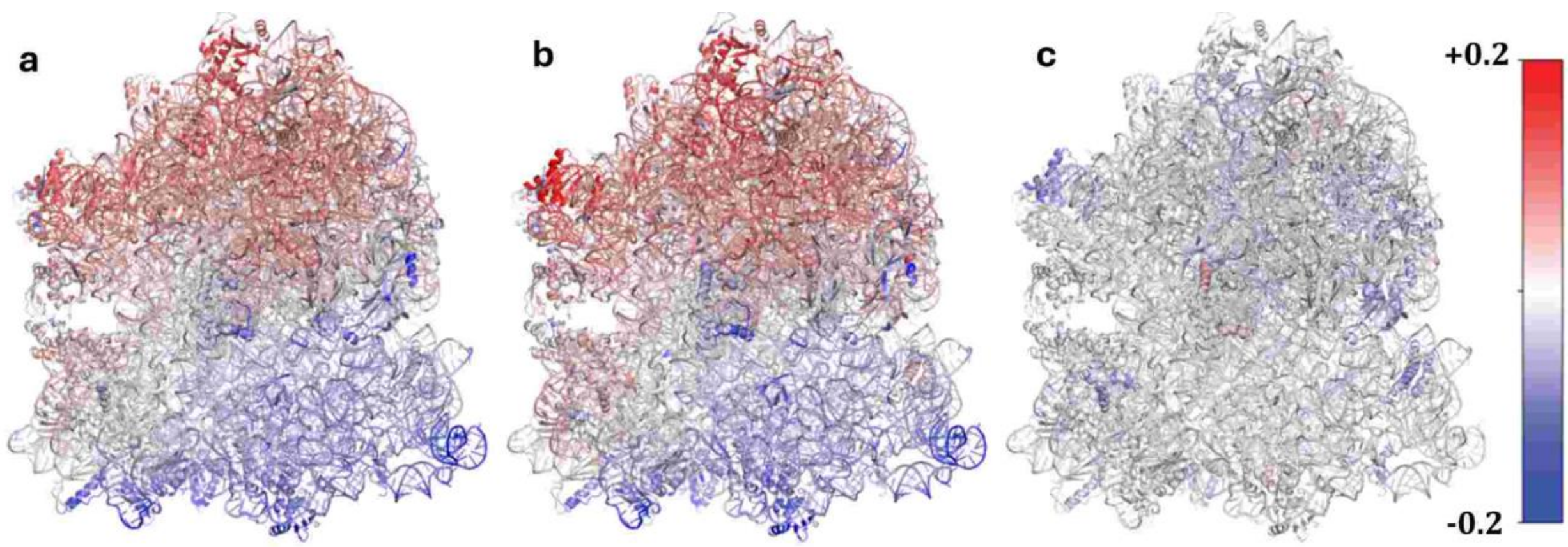

Fig. S2. *Robustness of ΔL11–WT DFI differences to alignment protocol.* ΔL11–WT differences in Dynamic Flexibility Index (DFI) computed using (a) whole-ribosome alignment, (b) separate alignment of the LSU and SSU, and (c) the difference between the two alignment approaches. While minor local deviations are observed, the overall spatial patterns and magnitudes of ΔL11–WT differences are preserved, indicating that the observed dynamical changes are not driven by alignment artifacts.

(2) As part of the Martini setup, the authors use an elastic network approach and then extract DFI and DCI which is based on linear response theory. Use of linear response is appropriate for small molecules, where the covariance matrix indeed relates to the Hessian as in eq (2). The free energy landscape of larger macromolecues and macromolecular complexes, however, has many local minima, which prevent straightforward application of linear response theory. Specifically, the covariance matrix contains all entropic contributions to the free energy, whereas the Hessian does not. As

the DCI is derived from DFI, it may suffer from the same flaws. To what extent their results depend on the parameter choices, e.g. the elastic network model? How well is the coupling described by the linear response?

We thank the reviewer for raising this important conceptual point regarding the applicability of linear response theory (LRT) to large macromolecular complexes. We agree that naïve application of Hessian-based harmonic approximations can be problematic for systems with rugged free-energy landscapes and multiple metastable states. We acknowledge that some of our original phrasing may have been ambiguous in this regard, and we thank the reviewer for pointing this out; the Methods section has been revised for clarity.

Importantly, our analysis does not rely on a Hessian-based formulation of LRT. Although DFI and DCI are conceptually motivated by LRT, the metrics themselves are computed entirely from the covariance matrix (CM) obtained from equilibrium sampling, without invoking a Hessian or assuming a single harmonic basin. In this formulation, the CM naturally incorporates both enthalpic and entropic contributions to the free energy landscape, as it reflects fluctuations sampled across the accessible conformational ensemble during the simulation.

LRT does not intrinsically require the use of a Hessian. Standard derivations based on Liouville's equation and perturbative expansions of the equilibrium distribution lead directly to response functions expressed in terms of the covariance matrix, provided the system is near equilibrium and perturbations are sufficiently small. This covariance-based formulation has been widely applied in biomolecular systems and does not assume a single quadratic minimum (Bretonnet, Statistical Mechanics for the Liquid State, 2020).

Within this framework, DFI may be viewed as a generalization of RMSF: while RMSF depends only on the diagonal elements of the CM, DFI incorporates the full covariance structure, thereby capturing correlated motions across the system. Although the diagonal terms dominate the magnitude of DFI, the off-diagonal elements encode long-range coupling and are essential for distinguishing allosteric from non-allosteric positions.

Finally, regarding parameter dependence, we note that the Go model is used only to stabilize the Martini representation and does not impose a harmonic free-energy landscape on the analysis itself. The covariance matrix is extracted from fully dynamical trajectories, and we observe that the qualitative DFI/DCI patterns are robust to reasonable variations in elastic network parameters (see Methods).

(3) The authors claim that “These findings suggest a dual effect of L11 deletion: it increases fluctuations in regions that normally require tight coordination during translation, while simultaneously restricting motions in regions that contribute to stress-induced structural transitions such as ribosome hibernation”. In my opinion this is an oversimplification since the decoding center is located on the SSU, a region with decreased fluctuations (according to Fig.1), and decoding requires a very tight coordination to monitor correct codon-anticodon base pairing.

We thank the reviewer for this thoughtful observation and agree that our original wording was overly broad. In particular, we agree that tight coordination is also essential at the SSU decoding center, where accurate codon–anticodon recognition is monitored through highly orchestrated structural rearrangements. (https://doi.org/10.1016/j.febslet.2013.05.009 and https://doi.org/10.1073/pnas.2004170117)

To address this, we have revised the text to avoid implying that “tight coordination during translation” is confined to the regions with increased fluctuations in our analysis. The revised sentence now reads:

“These findings suggest a dual effect of L11 deletion: it increases fluctuations in the PTC that normally require tight coordination during translation[37], while simultaneously restricting motions in regions that contribute to stress-induced structural transitions such as ribosome hibernation”

This revision is consistent with known ribosome function: catalysis at the PTC depends on tightly coordinated rearrangements that position substrates’ sites in precise geometry and control catalytic-site organization. (https://www.nature.com/articles/s41467-019-08880-0)

(4) Also, the comparison to the experiments does not provide a convincing validation of the computational results, because the experiments do not probe fluctuations or allostery in the ribosome; rather, they are, if at all, rather indirectly related to the presented computational results. E.g., the observed difference in sporulation behaviour and the differences of observed binding site dynamics could potentially be due to many other effects. E.g., the ΔL11 variant might simply lead to slower translation?

We thank the reviewer for this important point and agree that the experiments we compare to do not directly probe residue-level fluctuations or allosteric couplings within the ribosome. Our intention is not to claim direct experimental validation of the computed fluctuation profiles, but rather to evaluate whether the qualitative, system-level consequences predicted by the simulations are consistent with independently observed phenotypic outcomes.

As the reviewer correctly notes, the observed differences in sporulation behavior and binding-site dynamics could, in principle, arise from multiple mechanisms, including a global reduction in translation efficiency in the ΔL11 variant. However, a purely nonspecific slowdown in translation would be expected to affect germination, outgrowth, and sporulation in a largely similar manner, as all of these processes depend on protein synthesis capacity. In contrast, our results (Fig. 5) show that these processes are affected differentially, with sporulation efficiency remaining largely unchanged while spore revival (outgrowth) is significantly impaired. This divergence argues against a simple global translation-rate effect and is instead more consistent with process-specific regulatory perturbations.

Importantly, the role of L11 in translation and its impact on canonical translation factors (e.g., EF-Tu, EF-G) has been well established in prior genetic, biochemical, and structural studies, including effects on elongation dynamics and translation efficiency (e.g., Van Dyke et al., *J. Mol. Biol.* 2002; and subsequent structural/kinetic studies of the GTPase-associated center). We have revised the manuscript to incorporate these references and clarify that such global translation-related effects are already well characterized.

Within this context, our work focuses on a complementary and less explored question: how perturbations to L11 alter ribosomal dynamic coupling networks and whether such changes manifest in higher-level physiological processes. We therefore interpret the experimental results not as direct validation of specific allosteric pathways, but as independent, phenotype-level consistency checks that support the presence of nontrivial, pathway-dependent effects beyond a simple translational slowdown.

We have revised the manuscript to make this distinction explicit and to acknowledge that additional targeted experiments—such as direct measurements of translation rates or experimental probes of ribosomal dynamics—would be required to fully disentangle global translational effects from more specific dynamical and allosteric contributions.

(5) Assuming that changes in intersubunit rotation dynamics contribute markedly to the observed reduced SSU mobility, why should this affect dimerization? As the binding site does not involve the two subunits, I would think that the local flexibility of the binding site would have a larger effect on the binding than the dynamics of intersubunit rotation.

We appreciate this thoughtful question. While we agree that changes in intersubunit rotation dynamics could, in principle, influence SSU mobility, we do not believe that this effect alone can account for the observed differences in dimerization.

As noted in point (1), we additionally performed an analysis in which the LSU and SSU were aligned separately, thereby removing intersubunit rotation from the comparison.

This procedure did not reveal differences of sufficient magnitude to account for the observed effects.

Moreover, the 200-ns window length used in our analysis is substantially shorter than previously reported timescales for intersubunit rotation. For example, a computational study estimated a lower bound of approximately 1–5 ms for these motions (Freitas et al., 2021; https://doi.org/10.3390/biophysica1020016), and single-molecule FRET measurements report spontaneous rotational transitions on the tens-of-milliseconds timescale (Cornish et al., 2008; https://doi.org/10.1016/j.molcel.2008.05.004). Consequently, motions associated with intersubunit rotation are unlikely to be sampled within our 200-ns analysis windows.

(6) In the Methods section, the authors write that the structure was completed to account for proteins missing in the experimental structure. How was that done?

Missing proteins were added by structurally aligning appropriate supplemental ribosomal structures to our model and transferring the absent components. The resulting composite structures were subjected to brief energy minimization in vacuum, with position restraints applied to all heavy atoms, to remove any steric clashes introduced during the assembly process. For instance, proteins L10 and L11 were incorporated by superimposing the large subunit of PDB ID 3J9W onto our initial model, following an approach similar to that described in this study (https://doi.org/10.1016/j.bpj.2022.11.004)

(7) The authors have investigated the convergence of these observables by partitioning their trajectories into “overlapping windows”. Doesn’t this overlap compromise independence of the calculated observables and thereby lead to an underestimation of the s.e.m.? As the protocol of partitioning the data is not clearly described, I might be missing something, though.

We thank the reviewer for raising this important point regarding statistical independence. We apologize for the lack of clarity in our original description of the windowing protocol.

Although we stated that each trajectory was partitioned into overlapping windows of 200, 400, and 600 ns separated by a 200-ns lag, all results reported in the manuscript are based exclusively on non-overlapping 200-ns windows. These non-overlapping windows were used to compute the mean and standard error of the mean (s.e.m.), ensuring statistical independence between samples.

The 400-ns and 600-ns windows, which do involve overlap, were analyzed only as a convergence and robustness check, not for error estimation. Their purpose was to test

whether increased local sampling within longer windows altered the qualitative or spatial patterns of the observables by better capturing slow excursions or local minima.

We added supplemental figure S3, where we compare the DFI computed from non-overlapping 200-ns windows with that obtained from 400-ns windows, along with their difference. While minor deviations are observed in highly flexible peripheral regions, no differences occur in the ribosomal regions relevant to the analyses and conclusions presented in this work. Thus, overlap in the longer windows does not affect the reported observables or lead to an underestimation of uncertainty.

We have revised the Methods section to explicitly state that only non-overlapping windows were used for all reported statistics, and that overlapping windows were employed solely for convergence assessment.

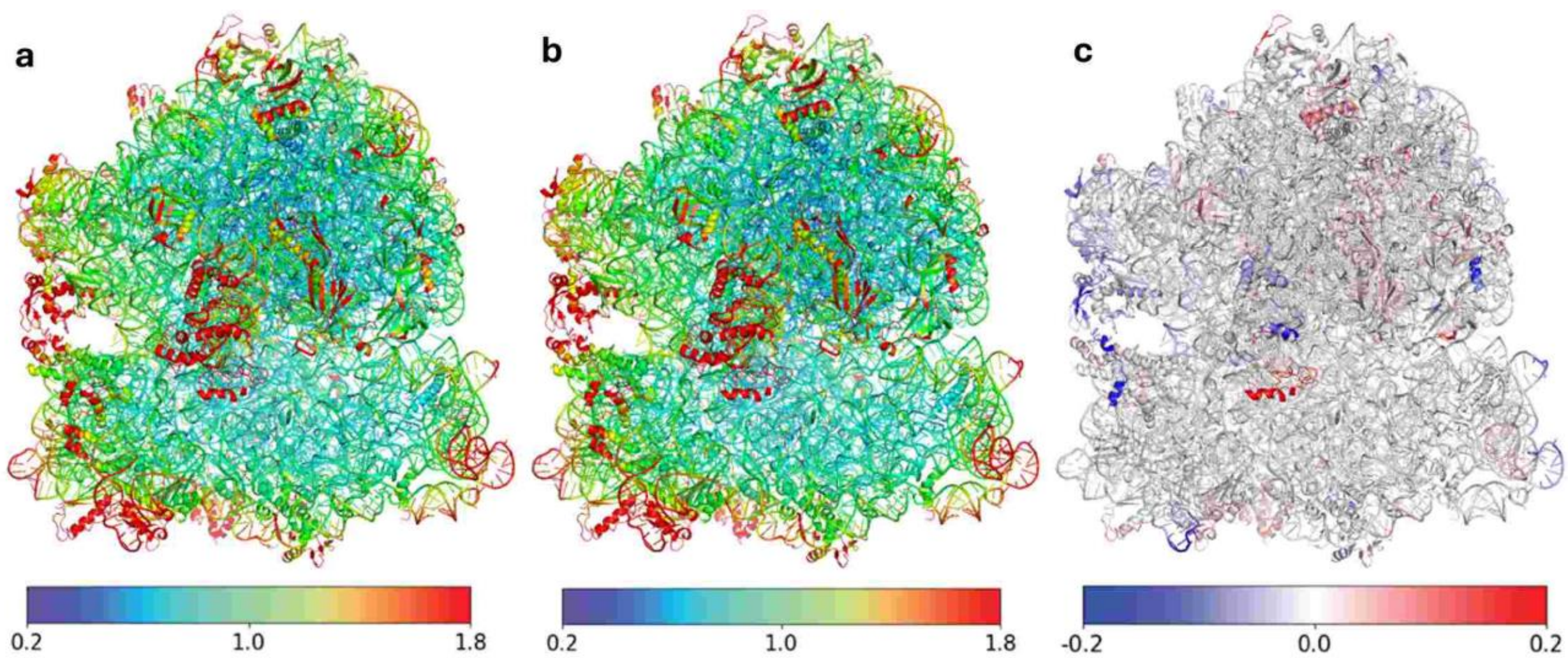

Fig. S3. *Robustness of convergence protocol.* We compare the DFI computed from non-overlapping 200-ns windows **(a)** with that obtained from 400-ns windows **(b)**, along with their difference **(c)**. While some deviations are observed in highly flexible peripheral regions, no differences occur in the ribosomal regions relevant to the analyses and conclusions presented in this work. Thus, the overlap in the longer windows does not affect the reported observations or lead to an underestimation of uncertainty.

(8) Is the coarse grained approach and the used force field accurate enough to support the conclusions? In a recent preprint, Cikhart et al. (https://arxiv.org/abs/2503.21357) tested the Martini (2.2) forcefield with several four different elastic networks and found substantial differences in the resulting dynamics. They also provided a direct comparison to all-atom MD simulations of the same length, showing that some elastic networks agreed with the all-atom simulations better than others. It would be instructive to compare the setup used here to the tested ones.

We appreciate the reviewer's question regarding the accuracy of the coarse-grained model and the relevance of recent benchmarking studies such as Cikhart *et al.*
During the development and validation of the force field used here, we performed direct comparisons against all-atom molecular dynamics simulations of comparable length. As one representative example, Figure 7 of our earlier work, Ref. [Biophysical Journal 2025, https://doi.org/10.1016/j.bpj.2025.07.034] demonstrates close quantitative agreement between backbone RMSF profiles obtained from our coarse-grained model and corresponding all-atom trajectories. This agreement holds across both structured cores and flexible regions, indicating that the essential fluctuation patterns are well captured.
The setups evaluated by Cikhart *et al.* are not directly transferable to the present work. In particular, our model differs substantially from Martini 2.2 in several key respects: (i) proteins are represented using a Go-like model rather than a generic elastic network, (ii) the RNA model is distinct from Martini 2.2, and (iii) additional force-field components—such as backbone dihedral potentials and protein–RNA coupling terms—play an important role in shaping the dynamics. Consequently, differences in elastic network parametrization alone do not provide a meaningful basis for direct comparison.
For these reasons, backbone RMSF was chosen as the primary validation metric, as it provides a robust, physically interpretable measure of dynamical fidelity and enables direct comparison between coarse-grained and atomistic simulations. The observed agreement supports the conclusion that the coarse-grained approach employed here is sufficiently accurate to capture the collective and mesoscale dynamics relevant to the analyses presented.
We have clarified this point in the revised manuscript to better delineate the scope of existing Martini benchmarks and the specific validation strategy used in this study.

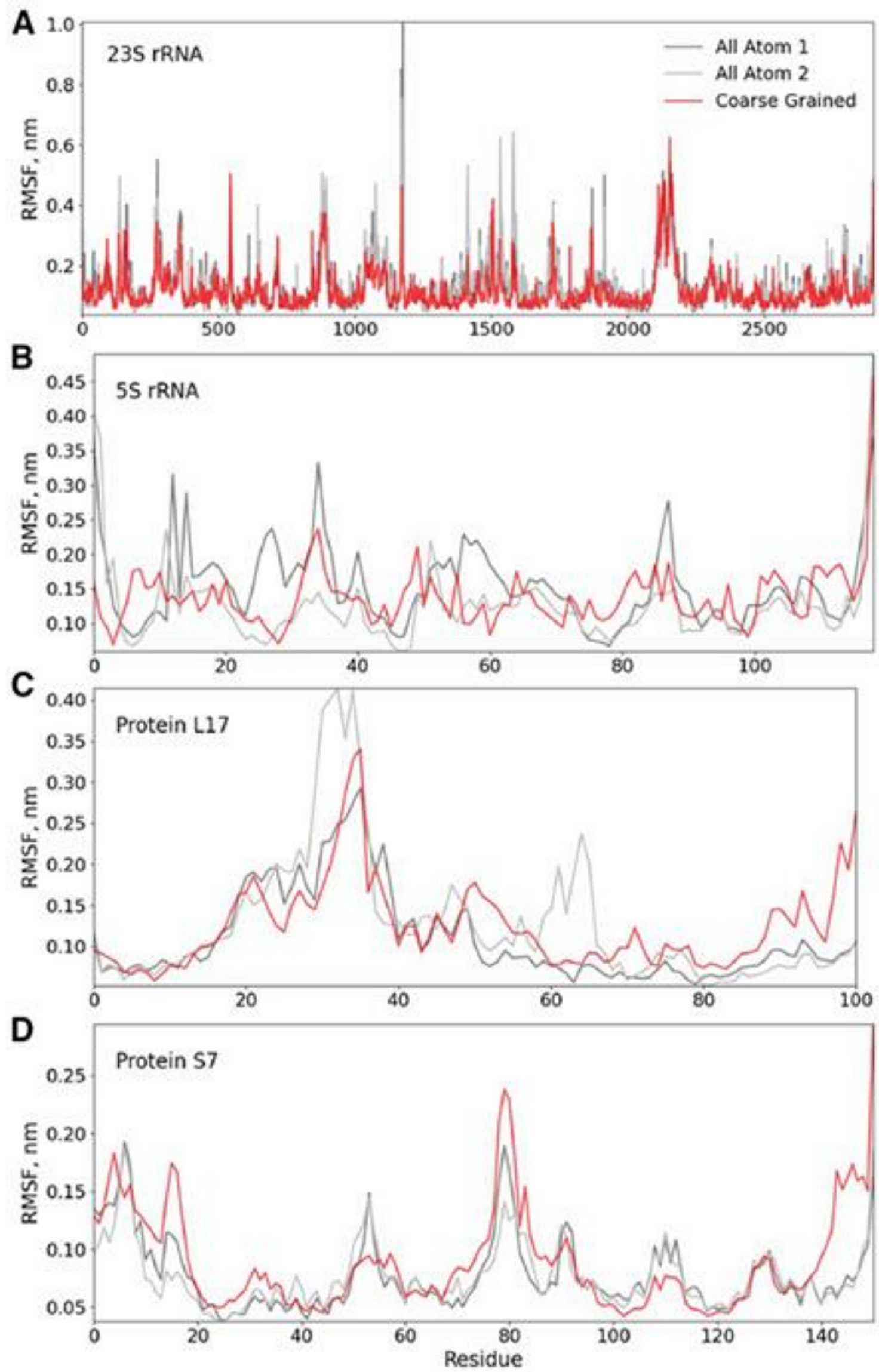

FIGURE 7 RMSF of rRNA and proteins in a 70S ribosome. Comparison between CG and AA RMSF for rRNA and two proteins that are in direct contact with it after 2 μs of MD simulations. CG Martini is shown in red, and two AA simulations are in gray and silver. (*A*) 23S ribosomal RNA. (*B*) 5S ribosomal RNA. (*C*) Large subunit protein L17. (*D*) Small subunit protein S7.

Minor concerns

(9) Several statements in the Introduction lack the supporting references:

We thank the reviewer for highlighting these omissions. The relevant references have now been added to the Introduction.

“… naturally occurring ΔL11 variant”
Galperin, M. Y., Wolf, Y. I., Garushyants, S. K., Vera Alvarez, R. & Koonin, E. V. Nonessential Ribosomal Proteins in Bacteria and Archaea Identified Using Clusters of Orthologous Genes. *J. Bacteriol.* **203**, (2021).

“Ribosomal protein L11 occupies a particularly strategic position in this context: located at the base of the L7/L12 stalk, L11 is a key communication node that couples factor binding, GTPase activation, and structural transitions across the large subunit.“
Dewachter, L. *et al.* The role of the essential GTPase ObgE in regulating lipopolysaccharide synthesis in Escherichia coli. *Nat. Commun.* **15**, 9684 (2024).

“Classical biochemical studies have long implicated L11 in recruiting translation factors and in regulating stress responses, ..”.
Walukiewicz, H. E. *et al.* Regulation of bacterial stringent response by an evolutionarily conserved ribosomal protein L11 methylation. *mBio* **15**, e01773-24 (2024).
Zhang, S., Scott, J. M. & Haldenwang, W. G. Loss of Ribosomal Protein L11 Blocks Stress Activation of the Bacillus subtilis Transcription Factor sB.

(10) “preserving the global collective modes necessary for this conformational adaptation”
Are these collective modes known? Is there a comparison?

We agree with the reviewer that, in many biomolecular systems, collective modes are often associated with the slowest, lowest-frequency motions and that their functional importance has been demonstrated in prior work. Indeed, previous studies have shown that slow collective modes can play a central role in large-scale conformational adaptation and allosteric regulation, including in ribosomal systems (e.g., https://www.pnas.org/doi/10.1073/pnas.1401864111; https://doi.org/10.1073/pnas.1632476100).

However, while these studies establish the importance of slow collective modes in ribosome function, they do not identify the specific modes associated with the conformational adaptation examined here, nor do they provide a direct basis for mapping individual modes onto the perturbations analyzed in our work. We therefore did not attempt an explicit mode decomposition or comparison, as this lies beyond the scope of the present study.

To reflect this distinction and avoid implying direct mode identification, we have adopted more cautious language in the manuscript. The revised text now states:

"Within this context, we hypothesize that L11 may contribute to allosteric stabilization of global collective modes involved in this conformational adaptation."

This formulation is consistent with established literature on the role of slow collective modes, while accurately reflecting the scope and limits of our analysis.

(11) "While these results are not a direct validation of our modeling results, they demonstrate that atomic scale studies of ribosome allostery […]" Coarse-grained simulations are not atomic scale.

We thank the reviewer for pointing out this imprecision. We agree that coarse-grained simulations do not constitute atomic-scale modeling. Our intention was to contrast the molecular level of description with cellular-scale phenomena, rather than to imply atomistic resolution.

To remove this ambiguity, we have revised the sentence to read:

"While these results are not a direct validation of our modeling results, they demonstrate that molecular-scale studies of ribosome allostery can potentially inform experiments at the cellular scale that would otherwise not have been obvious."

We believe this revised wording more accurately reflects both the level of resolution of our simulations and the scope of the conclusions drawn.

(12) "This increased rigidity extends across the interface responsible for 100S dimer formation, accompanied by a reduction in local mobility. In particular, the ΔL11 ribosome shows a pronounced decrease in vibrational entropy" In the Methods it is not clarified how local mobility was computed. Is it vibrational entropy? How was the vibrational entropy computed?

We apologize for the lack of clarity. By "local," we were referring specifically to motions near the HPF-binding interface, as opposed to the larger dimer formation interface, which partially overlap. We thank the reviewer for highlighting this point and have revised the text accordingly:

"Our analysis shows that deletion of ribosomal protein L11 leads to pronounced rigidification at the HPF-binding interface of the SSU (Fig. 3). This increased rigidity also extends into the region that contributes to 100S dimer formation. The resulting decrease in dynamical fluctuations—and consequently in vibrational entropy—is predicted to favor 100S dimer association and thereby enhance dimer thermodynamic stability[39–41]"

Reviewer #3 (Remarks to the Author):

In the manuscript „Ribosomal Allostery as a Regulator of Bacterial Dormancy” the authors apply ‘molecular dynamics simulations of wild-type and L11-deleted (ΔL11) ribosomes reveal that L11 functions as a global allosteric regulator coordinating communication between the ribosomal stalk and the peptidyl transferase center’.

Authors use sporulation in Bacillus subtilis as a model to test their working model. They discuss the role of L11 on the action of ribosomal hibernation factors HPF and RMF, proteins RelA and Obg.
It is surprising, the authors do not investigate what will happen to canonical translation factors such as EF-Tu or EF-G in the L11-deleted strain. Their function will be impaired, resulting in a strong phenotype.
Experiments presented in this manuscript do not support any of the claims of the authors and need to be redesigned. Some in vitro work would help support the model.
When is L11 absent from the ribosome? What fractions of the ribosome (wild type) would lack L11?
This manuscript is far from being acceptable for publication.

We thank the reviewer for raising important methodological and interpretational points regarding the molecular dynamics (MD) analysis and its connection to experimental phenotypes. We have revised the manuscript, added new analyses, and clarified the scope and interpretation of the results.

The reviewer expressed concern that MD-derived fluctuation and coupling metrics may not directly correspond to experimentally measurable quantities. We agree. Our goal is not to claim direct experimental validation of residue-level fluctuation profiles or specific allosteric pathways, which would require techniques such as NMR that are not feasible for intact ribosomes. Rather, our simulations are used to predict how global communication networks within the ribosome are altered by deletion of L11, and experiments are used to test whether the qualitative physical consequences of those perturbations are consistent with observable physiological outcomes.

We have revised the manuscript to clarify that experimental phenotypes are not intended to validate specific residue-level couplings, but rather to assess whether predicted large-scale perturbations in ribosomal dynamics are reflected in cellular behavior.

Importantly, we have also performed new analyses of our experimental data to show that the impact of L11 deletion on sporulation is distinct from its effect on spore revival (outgrowth). In particular while the timing play between WT ,and ΔL11 is comparable for

sporulation and outgrowth (approximately 6 hours), the efficiencies differ substantially: sporulation yield is nearly identical between WT and ΔL11, whereas spore revival efficiency in ΔL11 is only about one third of WT (see revised Fig. 5). *These effects are also distinct from the overall growth-rate differences observed during vegetative growth. This behavior is not consistent with a simple global reduction in translation efficiency, but instead suggests process-specific perturbations, consistent with altered ribosomal dynamic couplin*g.

We also agree with the reviewer that it is also critical to explore the role of L11 in translation in particularly the impact on canonical translation factors such as EF-Tu or EF-G in the L11-deleted strain. However, these roles have already been extensively explored. Classical genetic and biochemical studies have shown that limiting or deleting L11 alters translation termination efficiency and impairs growth, and that these effects are reversible upon restoring L11 expression (Van Dyke et al., *J. Mol. Biol.* 2002, DOI: 10.1016/S0022-2836(02)00304-2). Structural and kinetic studies of translation factor binding, including EF-Tu ternary complex accommodation, further establish the importance of the GTPase-associated center where L11 resides (ACS Chem. Biol. DOI: 10.1021/cb500409y). We have also revised our text to reflect these works and L11 deleted strains impact on canonical translation factors.

In summary, the importance of L11 for translation is well established. However, much of the detailed mechanistic work has focused on translational accuracy and elongation. In contrast, relatively little work has examined how perturbations of L11-mediated ribosomal dynamic coupling networks influence higher-level physiological state transitions, such as dormancy entry and exit.

Our study aims to bridge this gap by combining MD-based predictions of global ribosomal communication with phenotypic assays probing sporulation and revival. We have revised the Introduction and Discussion to better situate our work within this existing literature and to clarify that our contribution is not to redefine L11's canonical role in translation, but to propose and test a dynamic regulatory mechanism linking ribosome structure to stress-response physiology.

We also clarify that the ΔL11 strain used in this study is *a full deletion strain,* and therefore all ribosomes in that genetic background lack L11. We thus did not claim that wild-type cells naturally contain large fractions of L11-free ribosomes. Rather, we constructed the ΔL11 strain to serve as a *controlled perturbation* to interrogate how disruption of L11 affects downstream cell physiology. This distinction is now explicitly stated in the revised manuscript.

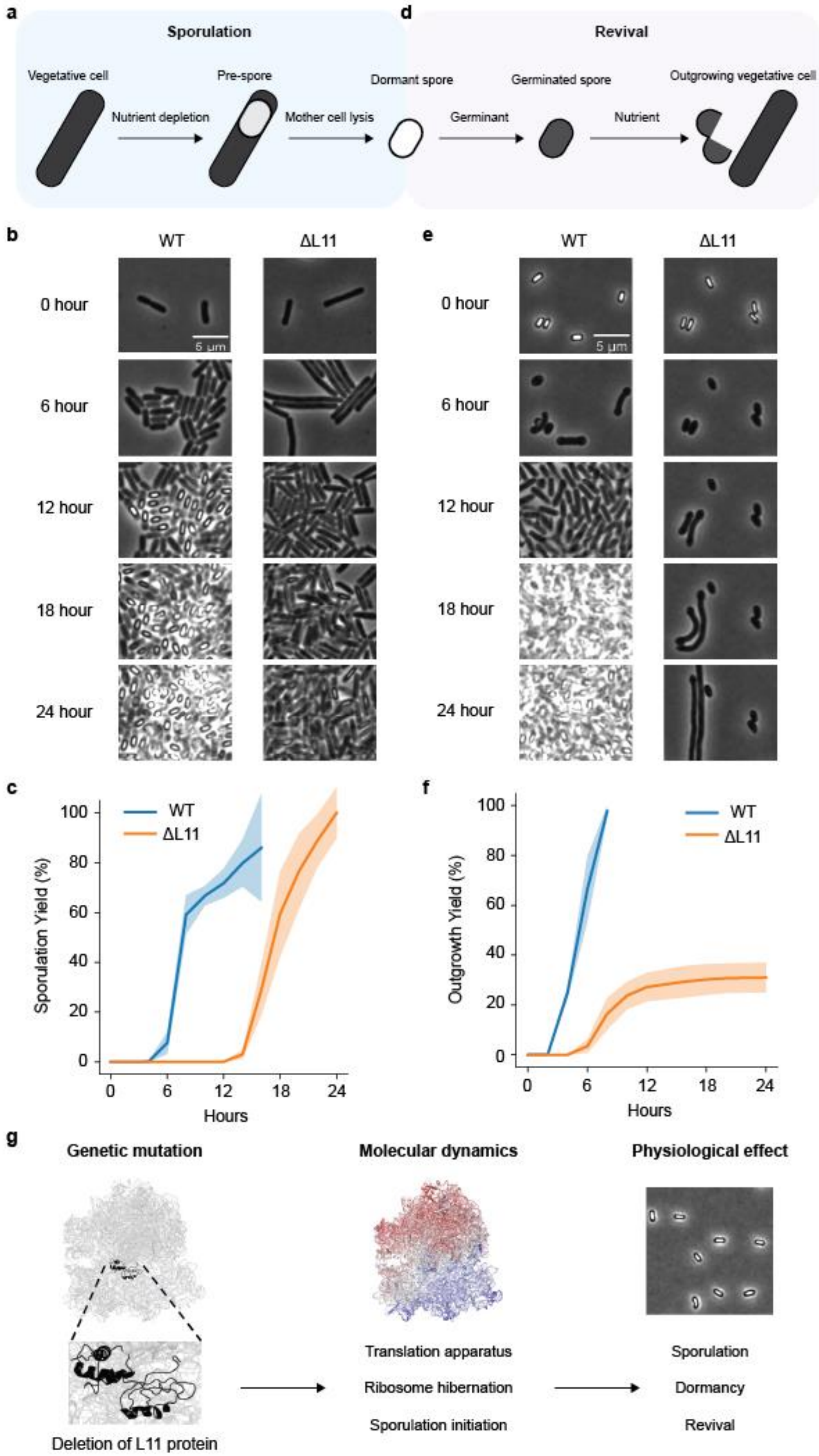

a
Sporulation
Vegetative cell
Nutrient depletion
Pre-spore
Mother cell lysis
d
Revival
Dormant spore
Germinant
Germinated spore
Nutrient
Outgrowing vegetative cell
b
WT
ΔL11
0 hour
6 hour
12 hour
18 hour
24 hour
5 μm
e
WT
ΔL11
0 hour
6 hour
12 hour
18 hour
24 hour
5 μm
c
Sporulation Yield (%)
Hours
WT
ΔL11
f
Outgrowth Yield (%)
Hours
WT
ΔL11
g
Genetic mutation
Deletion of L11 protein
Molecular dynamics
Translation apparatus
Ribosome hibernation
Sporulation initiation
Physiological effect
Sporulation
Dormancy
Revival

Figure 5. *ΔL11 is delayed in sporulation and impaired in spore revival.* (**a**) Schematic view of sporulation process. Under phase-contrast microscopy, the developing pre-spore becomes phase-bright within the phase-dark mother cell. (**b**) Phase-contrast microscopy filmstrips for representative WT and ΔL11 cells undergoing sporulation. Snapshots at 6-hour interval after cells were applied to an agarose pad composed of RM medium for inducing sporulation. (**c**) Sporulation yield, expressed as the number of spores formed per vegetative cell present at the time of sporulation induction and scaled to the maximum observed value across all conditions. Data were analyzed at 2-hour intervals (mean ± 95% confidence interval; n = 6 per strain). Because vegetative cells continue to replicate after induction, yields may exceed one spore per initial vegetative cell. (**d**) Schematic view of spore revival process. Spore phase-darkens during germination, followed by rupture of the spore coat and emergence of the outgrowing vegetative cell. (**e**) Phase-contrast microscopy filmstrips for representative WT and ΔL11 spores undergoing outgrowth. Snapshots at 6-hour intervals. (**f**) Outgrowth yield, expressed as the percentage of outgrowing spore, analyzed at 2-hour intervals (mean ± 95% confidence interval; n = 6 per strain). (**g**) Summary schematic of L11 ribosomal protein deletion. Loss of L11 is predicted to disrupt translational apparatus, hibernation state and sporulation initiation, leading to defects in spore formation and revival.

REVIEWER COMMENTS

Reviewer #1 (Remarks to the Author):

The authors have responded in appropriate ways to all of the points made in the referees' reports.

Reviewer #2 (Remarks to the Author):

The authors have markedly improved the manuscript and clarified several of my concerns. However, several other concerns remain that are not minor. Mainly the conclusions are still overstating the actually achieved confidence, the methods are in part not described to sufficient detail such that the study is reproducible, and the Introduction needs to more carefully reflect what is known and how this motivates the present hypothesis. I think these issues must and can be addressed. I'll list my comments below, following the numbering of my initial review.

(1) Based on the results of the test, I now agree that the particular type of structure alignment used does not appear to critically affect the results.

(2) The authors have sufficiently addressed this point. I agree it was mostly a matter of unclear writing, which is now clarified both in the reply as well as in the manuscript.

(3) The revision sufficiently addresses my point.

(4) Here I remain unconvinced. The remaining lack of a direct validation of the coarse-grained simulations via comparison to experiments renders it hard to judge how reliable the results are. In the previous paper about the development of the coarse-grained model, the model was compared to atomistic MD simulations using fluctuations. How well simulations of this model (which is the basis for the DFI and DCI calculations) can predict long-range allostery is not clear to me and needs to be directly checked against experiments for at least one of the presented mechanisms or for a similar combination of model and experiment. In my opinion, therefore, with the data at hand, the presentation of the conclusions is still too overconfident.

As an example, in the section “Rigidification of the small subunit and HPF binding interface”, based on the reduction in fluctuation at the 100S dimer interface upon removal of L11 observed in the simulations and ENN calculations of single and dimerized ribosomes, the authors suggest that 100S formation is favored for the ΔL11 variant. Based on this suggestion, they suggest that “ΔL11 ribosome may exhibit a delayed exit from dormancy”. Given the chain of uncertain inferences and the lack of discussion of alternative explanations, the final paragraph is an overstatement: “This observation further highlights the critical role of L11 in regulating the dynamic equilibrium between active and dormant ribosomal states, supporting its function as an allosteric inducer of translation and stress response pathways.”

As a further example, in the final paragraph of the Discussion, the authors state that “the higher affinity of ΔL11 ribosomes to form 100s dimers is consistent with a more stable dormant state that can explain delayed revival.”. However, the affinity was never measured or estimated, so stating the higher affinity as an established fact is not justified.

The authors need to adapt their wording of the conclusions to the actual strength or weakness of the evidence they provide.

We thank the reviewer for this critical feedback. We fully agree that our computational results based on a validated coarse-grained framework represent structural and dynamic predictions, rather than direct experimental measurements of thermodynamics or in vivo kinetics. Accordingly, we have revised the manuscript to more clearly distinguish between observations derived from the model and their biological implications, and to ensure that the latter are presented in a more cautious and appropriately qualified manner.

Specific changes to the manuscript:
1. We adjusted the wording and moved this to the next paragraph since they both discuss similar things.

While further experimental studies are required to determine if this translates to a delayed exit from dormancy in vivo, these dynamic shifts point toward a possible role for L11 in modulating ribosomal dynamics, influecing translation and stress response pathways.

2. We have replaced the term "affinity" (a thermodynamic property) with "increased structural rigidity," which are the physical properties directly measured by our DFI and DCI analyses.

In particular, the increased structural rigidity of 100s ΔL11 ribosome dimers is consistent with a more stable dormant state, providing a structural basis that may explain the observed delays in revival.

We note that prior studies have demonstrated correlations between changes in protein rigidity/dynamics and binding affinity in multiple systems—e.g., spike–antibody (https://doi.org/10.7554/eLife.92063) and peptide–WW domain interactions (https://doi.org/10.7554/eLife.67474). While these findings support the plausibility of such a connection, we emphasize that in the present work we do not directly quantify affinity, and therefore limit our conclusions to dynamical observations

(5) Here I disagree. While a full-scale intersubunit rotation is indeed not expected on the time scale of the simulations, in previous 100-ns long all-atom MD simulations (reference 10), rotations in the range of 5° were observed for several translocation intermediate states. Therefore, rotation in at least in this range would also be expected during the 200-ns long windows. I suggest the authors comment on this discrepancy or provide a reason for why this is not observed in their coarse-grained simulations.

We thank the reviewer for this insightful comment. We agree that previous all-atom simulations have reported inter-subunit rotations on the order of ~5° over comparable timescales, and we appreciate the opportunity to clarify this point. To address this, we performed a comparative analysis of the inter-subunit rotation for both our coarse-grained (CG) model and the all-atom (AA) model used in the initial RNA parametrization (see figures below).

To quantify the ratcheting angle, we aligned the Large Subunit (LSU) to a reference structure and computed the Kabsch matrix—the optimal rotation matrix that minimizes RMSD—for the Small Subunit (SSU) relative to the reference. The rotation magnitude was then derived from the trace of this matrix. For consistency, we excluded the first 200 frames and analyzed the subsequent 2,000 frames (representing 2 microseconds of simulation time).

Our results indicate that both models exhibit rotational dynamics that are quantitatively consistent with one another. While the observed rotations are approximately 2°, rather than the 5°, this difference likely stems from the specific definition of the rotation angle and our focus on the total magnitude of rotation.

Importantly, as confirmed by our separate subunit alignment analyses, the observed rotational fluctuations remain within a regime that does not affect the primary conclusions of our study, which are based on relative changes in dynamic coupling and

flexibility We have added a clarifying discussion in the manuscript to explicitly address this comparison and its implications.

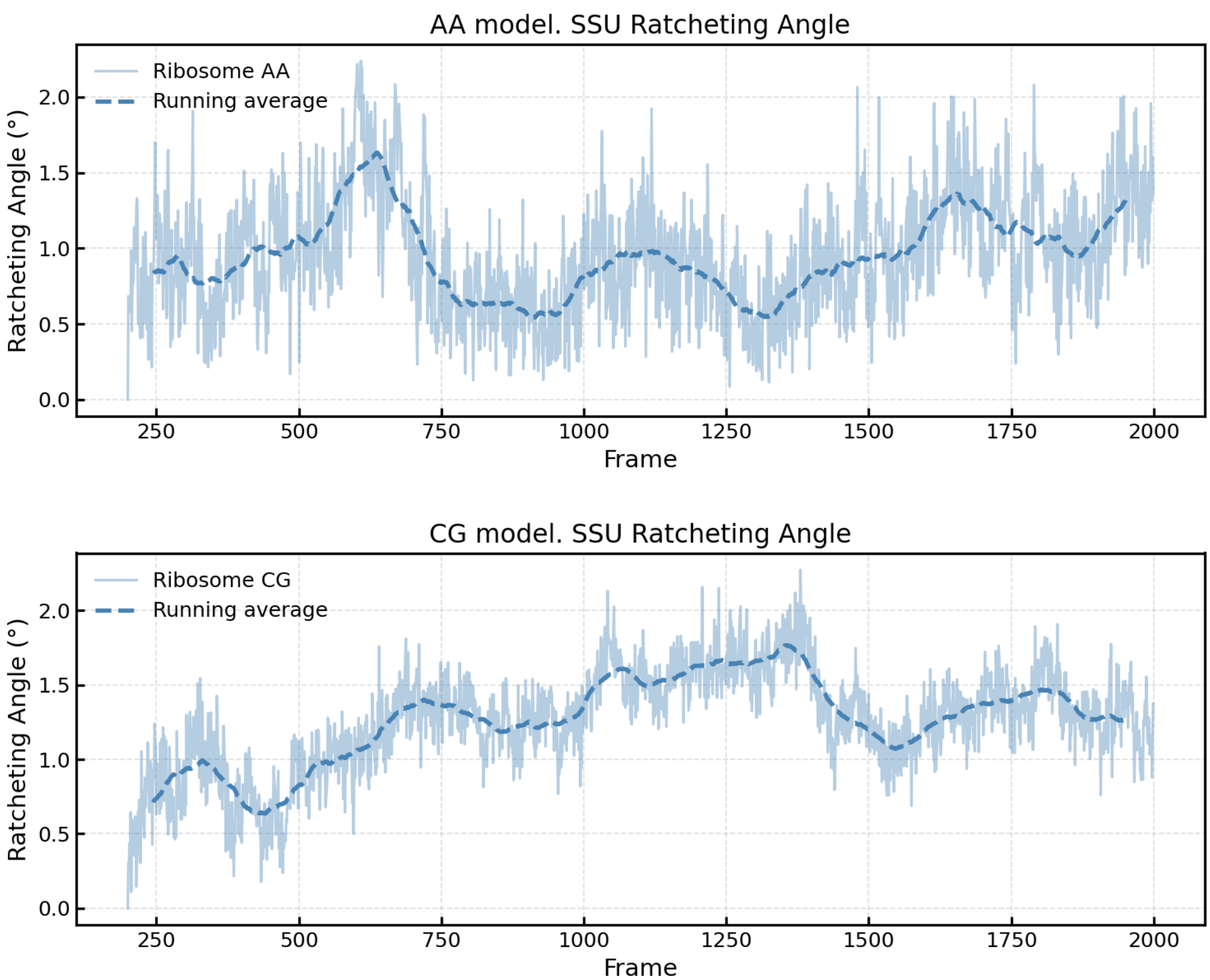

Figure 1. Comparison of inter-subunit rotational dynamics in all-atom(top)and our coarse-grained (bottom) ribosome simulations. The ratcheting angle of the Small Subunit (SSU) relative to the Large Subunit (LSU) was computed by aligning the LSU to a reference structure and extracting the optimal rotation of the SSU using the Kabsch algorithm. The rotation magnitude was obtained from the trace of the rotation matrix. Solid lines represent instantaneous angles, while dashed lines indicate running averages. Both AA and CG models exhibit comparable rotational fluctuations, with typical magnitudes on the order of ~2°, supporting the consistency of intersubunit dynamics across representations.

(6) Thanks for the explanation, but for the study to be reproducible, the manuscript (Methods) needs to contain the exact protocol of how the structure was complemented. Further, just telling that "appropriate supplemental ribosomal structures" were used is insufficient. The used structure (PDB 6HA1) is a cryo-EM structure of a ribosome containing a stalled peptide and an antibiotic. Are the stalled peptide and the antibiotic part of the simulated model? How would that specific complex affect the results?

We appreciate the reviewer's request for a more explicit protocol regarding our structural assembly. We have updated the Methods section to clarify the steps taken to construct the initial CG model, including the integration of missing proteins, ligands and the treatment of non-canonical residues. The updated section now reads:

Bacillus subtilis ribosome structure was derived from a cryo-EM model with a resolution of 3.1Å (PDB: 6HA1)[65]. All ligands, including antibiotics and the stalled peptide were removed from the initial structure. To complete the model, missing proteins L10 and L11 were incorporated by superimposing the large subunit of PDB: 3J9W[66] onto the initial structure. We then replaced the non-canonical nucleotides found within the experimental model by their chemically closest canonical counterparts to remain consistent with the available AA-to-CG mapping routines for the CG force-field. Finally, the resulting composite structures were subjected to brief energy minimization in vacuum, with position restraints applied to all heavy atoms, to remove any steric clashes introduced during the assembly process.

This procedure follows standard practices for preparing ribosomal systems for simulation, including removal of bound ligands not under study, completion of missing components using homologous structures, and adaptation of modified residues for compatibility with simulation frameworks (https://doi.org/10.1016/j.bpj.2022.11.004)

Because these non-ribosomal ligands (antibiotic and stalled peptide) were removed prior to model construction, they do not directly contribute to the dynamics reported here. As such, the simulations reflect the intrinsic behavior of the ribosome in the absence of these bound factors.

(7) The non-overlap of the windows is necessary but not sufficient for statistical independence. 200 ns is short compared to timescales of ribosomal motion (e.g. subunit rotation, see the above reply by the authors) and slow motions (a drift over longer timescales) could make the windows statistically dependent. The standard error of the mean would then underestimate the statical error (e.g. in Fig. 2b, d) and could lead to unsupported conclusions. The authors need to comment on this possibility.

In the Methods the authors then write "We then used the following procedure66 to assess convergence and errors: each trajectory was partitioned into windows of 200, 400, and 600 ns, each separated by a 200 ns lag, resulting in a total of n = 600 windows across all trajectories." And later "Finally, non-overlapping 200-ns windows were selected for analysis.", which should then be less than 600 windows. The Figure captions, however, say that n is 600. Which is correct?

We thank the reviewer for this important comment and agree that slow drift can lead to correlations between trajectory segments, even when windows do not overlap. We have revised the Methods section to explicitly acknowledge this point.

To assess the robustness of our results, we repeated the analysis using multiple window lengths (200, 400, and 600 ns), which sample different temporal regions and conformational states along the trajectories. As the windows progress in time, they naturally capture both local fluctuations and slower drift between states. The resulting DFI/DCI trends remained qualitatively consistent across window sizes and trajectory segments, indicating that the observed patterns are robust and not driven by a particular region of the trajectory or by long-timescale drift. We note that while correlations between trajectory segments may affect uncertainty estimates, the consistency across window sizes indicates that the reported trends are not sensitive to these effects.

Regarding the window count, the value n = 600 refers to the total number of non-overlapping 200-ns windows used in the final analysis across all replicates. With 12 independent replicas and 10,000 ns of production simulation per replica, this corresponds to a total simulation time of 120,000 ns, yielding 600 windows (12 × 10,000 ns / 200 ns = 600). We have clarified this calculation in the manuscript to avoid confusion.

The methods section now reads:
We then used the following procedure[75] to assess convergence and errors: each trajectory was partitioned into windows of 200, 400, and 600 ns, each separated by a 200 ns lag, resulting in a total of n = 600 (12 replicas * 10,000 ns / 200 ns) windows across all trajectories. While slow drift can lead to statistical dependence between windows, the consistent result between different window sizes effectively captures the relevant motions on ~200-400 ns timescale while minimizing the impact of long-term drift

(8) Thanks for the clarification.

(9) Regarding the new reference 27 (Dewachter, L. et al.), I don't see how this reference, which does not contain any structural information of the ribosome and does not even mention L11, supports the statement. Further, references 27 (Dewachter et al.) and 29 (Chakraborty et al.) are given as reference for the

sentence "Furthermore, GTPase Obg, a master regulator of sporulation in B. subtilis, binds near the base of the L7/L12 stalk in close proximity to ribosomal protein L11.". From the references, it appears that Obg binds to the isolated large subunit and prevents ribosome assembly. How does the role of Obg to prevent assembly relate to the presented work where allostery in the assembled ribosome is studied? Overall, the introduction needs to be markedly clearer in presenting what is known about the role of L11 and how the previous results relate to the hypothesized allostery. Currently, the introduction does not convincingly motivate the hypothesis of L11 being involved in allostery.

1. We thank the reviewer for this correction. The mismatch was due to an incorrect citation, which we have now resolved. We have revised the text to include appropriate references (27–30), along with DOI:10.1016/j.molcel.2008.01.009, which demonstrate that L11 plays a critical role in coordinating the L7/L12 stalk and regulating EF-G turnover. Importantly, we clarify that these studies establish L11 as a central functional element of the GTPase-associated center, motivating our hypothesis that it may also mediate long-range dynamical communication within the ribosome. We have revised the Introduction accordingly to avoid overstatement and to better distinguish known functional roles from the hypothesis explored in this work.
   The revised sentence now reads:
   Ribosomal protein L11 (or uL11 in universal nomenclature) occupies a particularly strategic position in this context: located at the base of the L7/L12 stalk, L11 is a key communication node that couples factor binding, GTPase activation, and structural transitions across the large subunit[27–30]

2. We thank the reviewer for pointing out the need for clarification regarding the role of Obg. We agree that the cited studies primarily describe Obg interactions with the large ribosomal subunit (LSU) and its role in ribosome assembly. We have therefore revised the text to avoid overstating this connection. Previous work (Feng et al., 2014; DOI:10.1371/journal.pbio.1001866) highlights that the role of the L7/L12 stalk in regulating Obg activity remains an open question. Notably, these conclusions are based on in vitro studies of the isolated LSU, and the available evidence remains limited. Accordingly, we now present this more cautiously and use this example only to emphasize that the L7/L12 stalk region, including L11, serves as an important interaction interface for translational GTPases. Consistent with this experimental context, we additionally analyzed the isolated 50S subunit using an Elastic Network Model and observed a qualitatively similar loss of dynamic coupling upon L11 deletion (Fig. S1).

   The revised section now reads:

As shown in Fig. 4b-d, the GTPase-associated center, also known as the factor binding center (FBC)[56–58], exhibits a dramatic loss of coupling to the ribosomal core upon L11 deletion. FBC acts as the primary docking station and molecular switch for the various GTPases that drive the translation cycle forward. One such GTPase, ObgE[31], plays a vital role in the onset of sporulation and the bacterial stringent response; thus the decreased dynamic coupling here may contribute to the impaired sporulation phenotypes observed in ΔL11 strain (Fig. 5). Feng et. al[33] previously described how GTPase-ribosome interactions induce conformational changes to propagate allosteric signals across multiple domains and explicitly noted that the potential role of the FBC in activating the ObgE GTPase remained an open question. Consistent with this, Fig. 4c reveals a substantial reduction in coupling between the FBC and distal regions, indicating disruption of long-range communication pathways upon L11 deletion. To ensure that this effect is not specific to the assembled 70S ribosome, we additionally analyzed the isolated 50S subunit and observed a qualitatively similar loss of dynamic coupling (Fig. S1)Ultimately, disruptions in these pathways, whether through the loss of ribosomal proteins or post-translational modifications, can significantly alter cell fate decisions under stress[59].

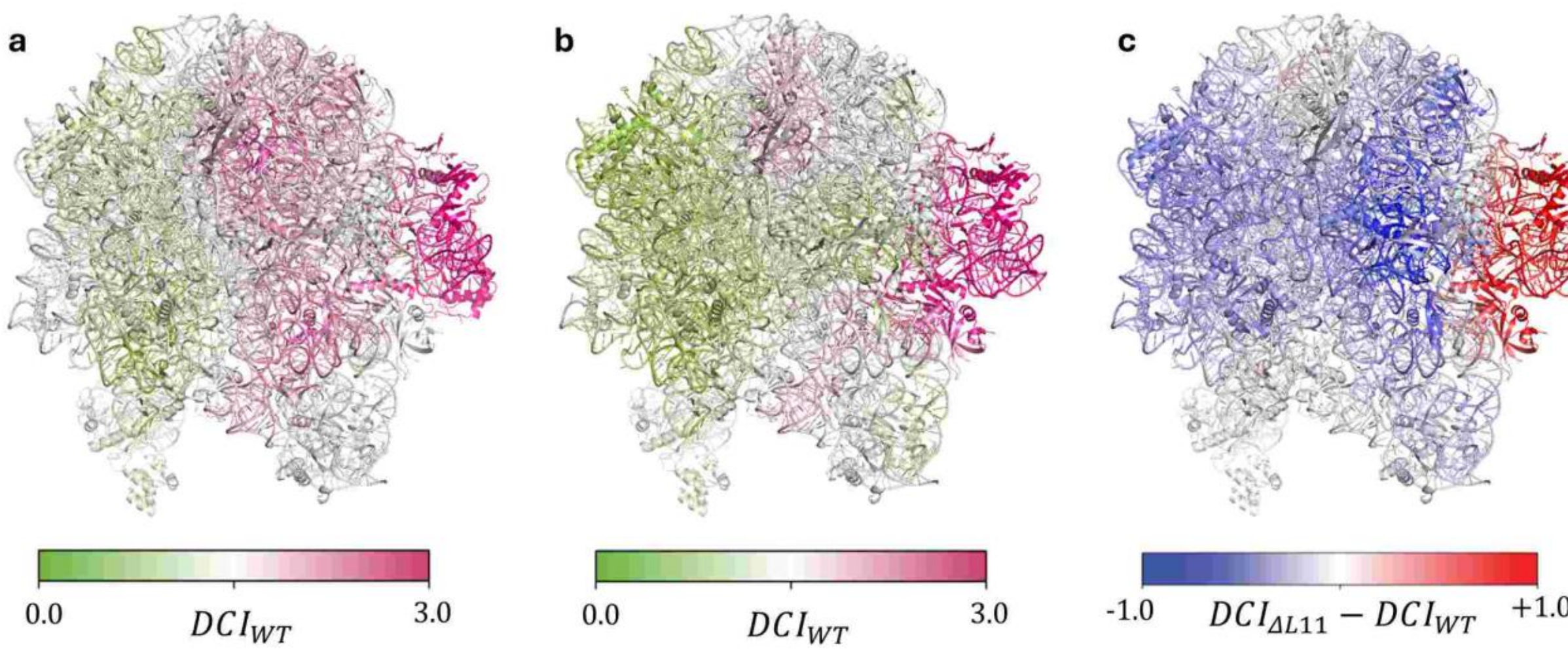

Fig. S1 *ENM analysis of the isolated 50S subunit in wild-type and ΔL11 systems*. (a) DCI between the factor-binding center (FBC) and the remainder of the 50S subunit in the wild-type, (b) the same analysis for ΔL11, and (c) the change in DCI upon L11 deletion, highlighting a loss of dynamic coupling within the large subunit.

The remaining minor comments have been sufficiently addressed.

One additional minor comment: The bacterial protein L11 corresponds to protein L12 in yeast and human ribosomes. To avoid confusion, I suggest to

either switch to or at least mention the commonly used name uL11 (see https://doi.org/10.1016/j.sbi.2014.01.002 for the nomenclature).

We appreciate this suggestion regarding the ribosomal protein nomenclature. We have updated the manuscript to include the "uL11" designation (as per Ban et al., 2014) alongside "L11" to ensure clarity and consistency with current naming standards.

> Ribosomal protein L11 (or uL11 in universal nomenclature) occupies a particularly strategic position in this context: located at the base of the L7/L12 stalk, L11 is a key communication node that couples factor binding, GTPase activation, and structural transitions across the large subunit[27–30]

Reviewer #3 (Remarks to the Author):

I have some additional questions and requests for the authors.
How is sporulation efficiency calculated? Pictures provided for mutant cells do not look like nearly 100% spores.

We thank the reviewer for raising this important question. Sporulation efficiency in our study was defined as the number of spores formed per vegetative cell present at the time of sporulation induction. Specifically, we calculated sporulation yield as the ratio of spores to the initial number of vegetative cells, normalized to the maximum yield observed under any condition in the experiment (set as 100%). This information is described in the corresponding figure caption and Methods section.

Since vegetative cells continue to divide after the induction of sporulation, and the ΔL11 mutant undergoes more replication cycles than WT, the proportion of ΔL11 spores at any given time point may seem to be lower than that in wild-type populations. However, the overall yield (spores per starting cell) is as reported in Figure 5.

While the results of the experiment presented are interesting and clearly show differences compared to the wild-type Bacillus strain, they are insufficient to support the statements in the manuscript.
What happens to the ribosomes in the mutant cells? The effect on hibernation and RNA degradation can be tested. Authors should check ribosomes using sucrose gradient centrifugation and the integrity of rRNA by isolating the RNA and testing it on a gel or by gel capillary electrophoresis. Observed differences in sporulation efficiency and germination could be a secondary effect. The authors should show the direct link to ribosome hibernation and/or degradation.

Why are Bacillus cells not recovering after germination? Even after 24 hrs they are not dividing and they do not re-enter sporulation.

We appreciate the reviewer's thoughtful comments and agree that examining ribosome hibernation and rRNA integrity would further strengthen the mechanistic interpretation. However, our current study was designed to probe the functional and dynamic consequences of L11 deletion at a systems level, rather than to re-characterize the well-established structural or biochemical roles of L11 in ribosome stability. We have revised the Discussion to make this scope clear and to outline how these additional measurements could serve as valuable follow-up experiments in future studies aimed at dissecting ribosome hibernation or degradation mechanisms.

To further explore the broader physiological consequences of L11 deletion beyond translation and specifically to test possible allosteric impacts of L11, we have now examined cell morphology as another systems-level indicator of altered regulation. We find that L11 cells are elongated, which aligns with known effects of (p)ppGpp depletion on cell division. This finding supports our mechanistic model linking perturbation of ribosomal allostery through L11 to interfere with stress-response signaling at the distant RelA binding site. Notably, this unusual morphology appears to persist even in the spore form. To our knowledge, this is a novel phenotype, as no other ribosomal mutation has been reported to produce elongated spores. This new observation that further strengthens our claim that L11 is a central allosteric modulator of ribosomal function as a signaling component that extends beyond its role in translation. This data and analysis are now included in the manuscript:

Morphology defects in ΔL11

We next examined cell morphology as another systems-level indicator of altered cellular regulation by the L11 deleted ribosome and its impact on stress-induced signaling. Consistent with prior studies linking L11 mutation to impaired RelA activation and reduced (p)ppGpp synthesis (PMID: 11673421, 4610577), ΔL11 cells display pronounced elongation (Fig. 5g). This phenotype parallels the known effects of (p)ppGpp depletion on cell septation (PMID 26644431) and therefore supports our mechanistic model linking perturbation of L11 that allosterically alters ribosomal stress-response signaling. Notably, this elongated cell morphology also persists in the spore form: ΔL11 spores are longer in length compared to WT spores (Fig. 5h), suggesting that the underlying defect in septation becomes structurally embedded during sporulation. This persistent elongated morphology highlights a previously unrecognized phenotypical consequence of L11 deletion that highlights it allosteric importance.

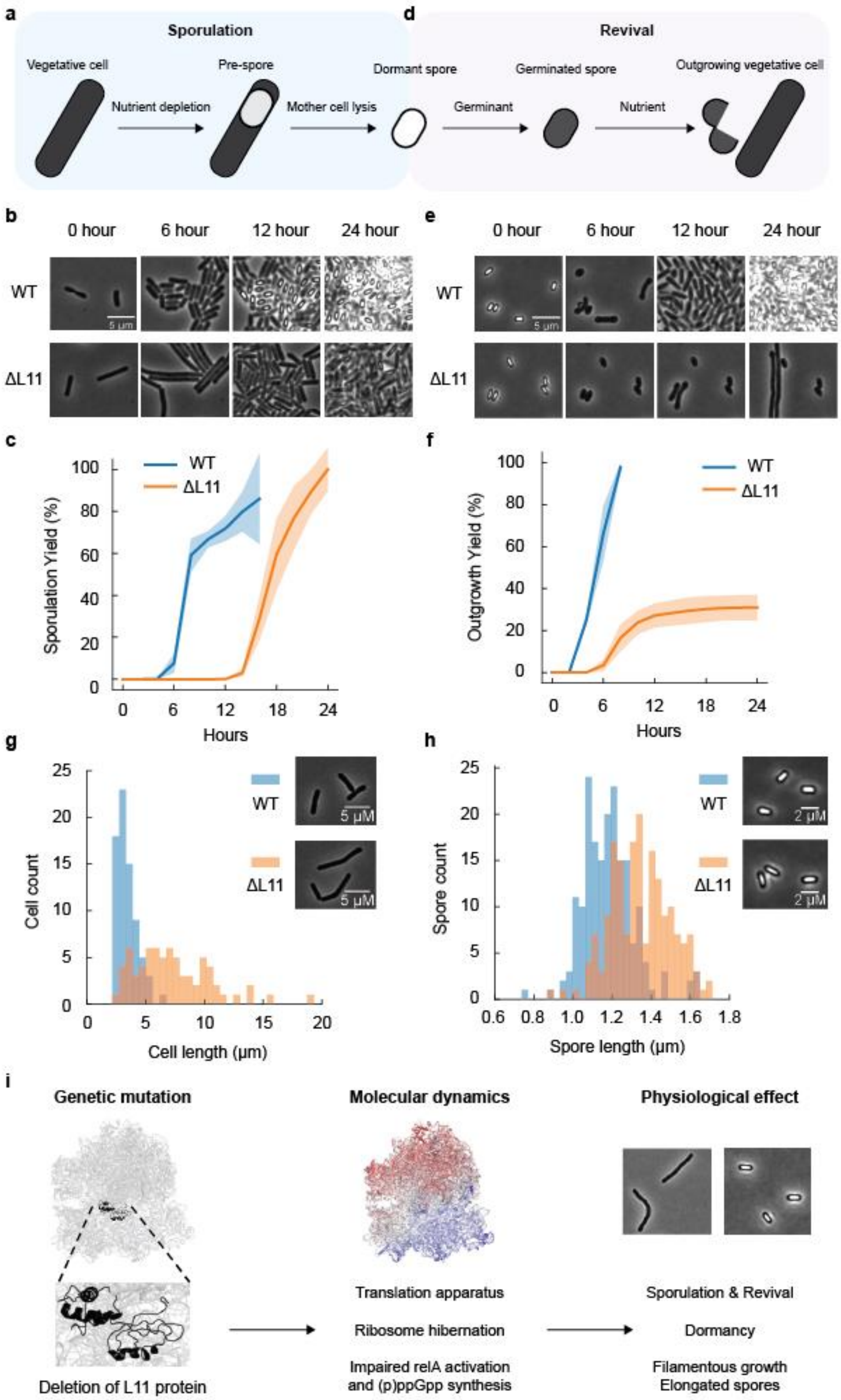
a
Sporulation
Vegetative cell
Nutrient depletion
Pre-spore
Mother cell lysis
d
Revival
Dormant spore
Germinant
Germinated spore
Nutrient
Outgrowing vegetative cell
b
0 hour
6 hour
12 hour
24 hour
WT
ΔL11
e
0 hour
6 hour
12 hour
24 hour
WT
ΔL11
c
Sporulation Yield (%)
Hours
WT
ΔL11
f
Outgrowth Yield (%)
Hours
WT
ΔL11
g
Cell count
Cell length (μm)
WT
ΔL11
5 μM
h
Spore count
Spore length (μm)
WT
ΔL11
2 μM
i
Genetic mutation
Deletion of L11 protein
Molecular dynamics
Translation apparatus
Ribosome hibernation
Impaired relA activation and (p)ppGpp synthesis
Physiological effect
Sporulation & Revival
Dormancy
Filamentous growth
Elongated spores

Figure 5. Physiological consequences of *ΔL11 in sporulation, revival, and morphology.* (**a**) Schematic view of sporulation process. Under phase-contrast microscopy, the developing pre-spore becomes phase-bright within the phase-dark mother cell. (**b**) Phase-contrast microscopy filmstrips for representative WT and ΔL11 cells undergoing sporulation. Snapshots at 6-hour interval after cells were applied to an agarose pad composed of RM medium for inducing sporulation. (**c**) Sporulation yield, defined as spores formed per vegetative cell present at the time of sporulation induction and scaled to the maximum observed value across all conditions. Data were analyzed at 2-hour intervals (mean ± 95% confidence interval; n = 6 per strain). Because vegetative cells continue to replicate after induction, yields may exceed one spore per initial vegetative cell. (**d**) Schematic view of spore revival process. Spore phase-darkens during germination, followed by rupture of the spore coat and emergence of the outgrowing vegetative cell. (**e**) Phase-contrast microscopy filmstrips for representative WT and ΔL11 spores undergoing outgrowth. Snapshots at 6-hour intervals. (**f**) Outgrowth yield, expressed as the percentage of outgrowing spore, analyzed at 2-hour intervals (mean ± 95% confidence interval; n = 6 per strain). (**g**) Histogram of WT and ΔL11 cell length (n = 75 cells per strain). Snapshots of representative cells included on the right. (**h**) Histogram of WT and ΔL11 spore length (n = 73 spores per strain). Snapshots of representative spores included on the right. (**i**) Summary schematic of L11 ribosomal protein deletion. Loss of L11 is predicted to disrupt translational apparatus, hibernation state and stringent response, leading to defects in entry and exit from dormancy, and cell and spore morphology.

Regarding the reviewer's question about why ΔL11 cells fail to recover after germination, this observation is in fact central to our main conclusion. The prolonged post-germination dormancy we observe is consistent with the hypothesis that L11 influences cellular processes beyond its canonical role in translation. While L11 deletion is known to impair translation efficiency and growth, such global effects alone would not be expected to maintain cells in a metabolically quiescent state for more than 24 hours after germination. Instead, the persistence of non-growing, non-dividing cells suggests a deeper failure to re-engage the regulatory programs required for re-entry into active growth. We have clarified this in the revised Discussion, emphasizing that the extended dormant phase supports our proposal that perturbations to ribosomal dynamic coupling interfere with physiological state transitions, rather than simply slowing translation.

> Many ΔL11 spores remain in a non-growing, non-dividing state for more than 24 hours after germination. This prolonged post-germination dormancy suggests a deeper failure to re-activate the regulatory programs required for re-entry into growth. Together, this behavior is not consistent with a simple global reduction in translation efficiency but instead indicates process-specific perturbations arising from altered ribosomal long-range dynamic coupling.

REVIEWER COMMENTS

Reviewer #2 (Remarks to the Author):

The authors have now addressed all my comments satisfactorily.
We thank the reviewer for careful comments and suggestions throughout the review process.

Reviewer #3 (Remarks to the Author):

The authors of the manuscript „Ribosomal Allostery as a Regulator of Bacterial Dormancy" did not provide convincing experimental data supporting their hypothesis.

Deletion of L11 ribosomal protein will affect the entire proteostasis of the sporulating Bacillus subtilis. The authors do not show any evidence directly pointing the loss of protein with dormancy of Bacillus. Impaired sporulation can be linked to defective translation, since deletion of L11 will also affect canonical translation factors such as EF-G and EF-Tu. It is known that perturbations to the ribosome such as deletion of ribosomal proteins, can affect maturation of spores. For example deletion of L31 protein as shown by Sinai et al PMID: 25661487 or paralogs of L31, L33 and S14 as shown by Iwanska et al PMID: 39169056 show defects to the spore maturation and dormancy. The authors of this manuscript are not showing what happens to the ribosomes. There is no in vitro monitoring of the translation (AHA, OPP PMID: 39753535, PMID: 39169056), no sucrose gradient polysome profiling (formation of 100S), monitoring of rRNA degradation (PMID: 22209493), no direct evidence. Translation in sporulation is fairly well described (PMID: 39753535, PMID: 39169056) and so is germination (PMID: 25661487). The authors do not show what happens to the ribosomes in their experiment, either in vivo or in vitro.

The experiments presented in this paper show the consequences of the impaired proteostasis of the cell. And we observe indirect consequences, secondary effects. To confirm the hypothesis, the authors should show direct evidence.

The authors claim that RelA action and ppGpp synthesis are affected. But they still observe spore formation. They should show that indeed ppGpp synthesis is abolished. Moreover, inhibition of ppGpp synthesis during sporulation should result in no spore phenotype (Wexselblatt et al PMID: 23028324), which is not the case in the presented work.

The elongated phenotype of L11 also does not explain the hypothesis. Again, they show no direct evidence, no localization of translation, and no septation (which could be done using a membrane stain).

The experimental part needs to be redesigned and redone.

We appreciate these comments. While we did not experimentally test every hypothesis generated by our modeling, our experiments were specifically chosen to evaluate whether the predicted changes in the dynamics of the ribosome manifest as observable effects on bacterial sporulation and dormancy. We have revised the manuscript to more clearly distinguish between our computational predictions and biological observations, ensuring that our conclusions are appropriately qualified. Accordingly, we have now toned down our claims regarding the experimental support of our model. This work is primarily computational, and the experiments are intended to provide supporting context rather than a direct test of the modeling predictions. In particular, we do not aim to directly experimentally validate atomic-scale predictions, as this would be beyond the scope of the present study. We have revised the text to clarify that the main objective is to present the computational framework and its results.

The following are the edits made for the submission:

1. Overall, these results highlight the role of L11 in ribosomal allostery, suggesting how local perturbations propagate through the ribosome to influence global physiological outcomes and bacterial survival under environmental stress.

2. We find that both sporulation and outgrowth are impaired, and together, these results suggest that dismantling a single ribosomal hub not only collapses ribosome-scale allostery but may also compromise survival strategies that span cellular scales, pointing to a previously unrecognized potential role for ribosomal regulation in sporulation.

3. Taken together, these findings potentially provide a mechanistic rationale for the impaired sporulation in the ΔL11 variant.

4. **Morphology defects in ΔL11.** We next examined cell morphology as another systems-level indicator of altered cellular regulation by the L11 deleted ribosome and its connection on stress-induced signaling. Consistent with prior studies linking L11 mutation to impaired RelA activation and reduced (p)ppGpp synthesis[64,65], ΔL11 cells display pronounced elongation (Fig. 5g). This phenotype is similar to the known effects of (p)ppGpp depletion on cell septation[66] and supports a linking between L11 perturbation and altered ribosomal stress-response signaling. Notably, this elongated cell morphology also persists in the spore form: ΔL11 spores are longer in length compared to WT spores (Fig. 5h), suggesting that the underlying defect in septation becomes structurally embedded during sporulation. The elongated spores represent a novel phenotypic effect of L11 deletion, consistent with its potential role in allosteric signaling.

5. These effects are qualitatively consistent with the reduced 100S dimerization propensity and altered coupling at hibernation, GTPase and RelA interfaces suggested by the simulations

6. Together, this behavior is not fully explained by a simple global reduction in translation efficiency, and raise the possibility of process-specific perturbations associated with altered long-range ribosomal dynamic coupling.

7. The absence of L11 may compromise efficient ribosome hibernation, potentially increasing susceptibility to degradation and thereby affect the energetic economy required for rapid reactivation.

8. Together, these results suggest that L11 contributes to the allosteric regulation of ribosome function. Our data indicate that L11 facilitates long-range communication between distant sites—including RelA, HPF, and the PTC—through pathways of allosteric dynamic coupling. Loss of L11 appears to disrupt these interactions, impacting both translational efficiency and the capacity for adaptive persistence. Importantly, our findings echo a broader theme across cellular systems: that rigidity and flexibility must be precisely balanced for macromolecular machines to operate efficiently and adaptively[70]. In this context, L11 serves as a node that tunes the ribosome's mechanical landscape, supporting the transitions between active translation and protective dormancy.
